# Building trust in deep learning-based immune response predictors with interpretable explanations

**DOI:** 10.1038/s42003-024-05968-2

**Published:** 2024-03-06

**Authors:** Piyush Borole, Ajitha Rajan

**Affiliations:** School of Informatics, University of Edinburgh, Informatics Forum, 10 Crichton St, Newington, Edinburgh, EH8 9AB Scotland UK

**Keywords:** Machine learning, MHC class I

## Abstract

The ability to predict whether a peptide will get presented on Major Histocompatibility Complex (MHC) class I molecules has profound implications in designing vaccines. Numerous deep learning-based predictors for peptide presentation on MHC class I molecules exist with high levels of accuracy. However, these MHC class I predictors are treated as black-box functions, providing little insight into their decision making. To build turst in these predictors, it is crucial to understand the rationale behind their decisions with human-interpretable explanations. We present MHCXAI, eXplainable AI (XAI) techniques to help interpret the outputs from MHC class I predictors in terms of input peptide features. In our experiments, we explain the outputs of four state-of-the-art MHC class I predictors over a large dataset of peptides and MHC alleles. Additionally, we evaluate the reliability of the explanations by comparing against ground truth and checking their robustness. MHCXAI seeks to increase understanding of deep learning-based predictors in the immune response domain and build trust with validated explanations.

## Abstract (web summary)

This study aims to build trust in MHC class I predictors by providing human-interpretable explanations. The explanations were assessed for accuracy, consistency across prediction models, and stability with similar peptides.

## Introduction

The Major Histocompatibility Complex (MHC) class I pathway supports the detection of cancer and viruses by the immune system. It presents parts of protein (peptides) from inside a cell on to the membrane surface enabling visiting immune cells that detect non-self peptides to terminate the cell. The ability to predict whether a peptide will get presented on MHC class I molecules is a key component in vaccine design since it helps determine if the vaccine can activate the immune system to destroy the invading pathogen. Numerous deep learning (DL)-based models for predicting peptide presentation on MHC class I molecules have emerged, demonstrating high prediction accuracies in predicting presented peptides. The deep learning models in the MHC class I predictor literature have a wide variety of architectures - Multilayer Perceptron (NetMHCpan-4.1^1^), Convolutional Neural Networks (MHCfovea^2^, ConvMHC^3^), Transformers (TransPHLA^4^, ACME^5^), Gated Recurrent Unit neural networks (MHCSeqNet^6^), etc. However, owing to the inherent inscrutability of deep learning models, it is difficult to understand and interpret predictor performance for the peptide-MHC allele instances and rationalize the differences observed between the predictors. Consequently, this raises the question of whether we can trust these deep learning MHC class I predictors.

Holzinger^7^ emphasized the importance of two fundamental aspects for trustworthy AI: robustness and explainability. These two pillars are also highlighted in the ethical guidelines for trustworthy AI by the European Commission^8,9^. Robustness of an AI model refers to the ability of a model to maintain its performance when faced with uncertainties or adversarial conditions. This includes handling noisy data, distribution shifts, and adversarial attacks, among other challenges. On the other hand, Explainability or Explainable Artificial Intelligence (XAI) focuses on enhancing the transparency and understandability of AI model decisions and predictions for end-users. XAI techniques have been proposed in recent years to help explain and understand the output of deep learning models used in image classification^10–16^ and NLP classification tasks^17–21^. There is, however, limited work on explainability and interpretability for deep learning-based MHC class I predictors. This article concentrates on explainability as a means to enhance trust in AI models. While robustness is equally important, it is considered a subject for future exploration in our work.

Related work for MHC class I predictors largely focus on global explainability, that is trends and explanations observed across the whole input dataset, rather than local explainability focusing on individual input instances. ACME and TransPHLA use attention scores to provide both global explanation and explanation for just one input (Instance-based or local explanation). However, the use of attention scores as an explanation is not reliable^22^ and is architecture specific, making it unusable for most MHC class I predictors that use other architectures as Convolutional Neural Networks or Multilayer perceptron. PoSHAP, proposed by^23^ in 2022, is related to the interpretations that we use. However, PoSHAP in their contribution only consider the Long Short-Term Memory (LSTM) deep learning architecture and focus their analysis on producing global, rather than local, explanations. Our work focuses on post-hoc explanations, i.e., explanations for an existing model that has been previously trained, which we treat as a black-box system. Such post-hoc explanations are widely applicable as they can be used over predictors whose internal structure is not known.

As a first contribution in this article, we use two popular XAI techniques, Locally Interpretable Model Agnostic Explanations (LIME)^10^ and SHapley Additive exPlanation (SHAP)^24^, from the image classification domain to interpret the outputs from MHC class I predictors. Both XAI techniques are model agnostic and can be applied to any deep learning-based MHC class I predictor, irrespective of architecture. The fundamental idea behind LIME is to create understandable explanations for complex machine learning models by approximating their behavior in a local area using a simpler, interpretable model. This is done using the following steps, 1. Select an instance for which an explanation is needed, 2. perturb the instance by introducing slight variations or noise, 3. predict the output for the perturbed instance using the black-box complex model. Repeat Steps 2 and 3 for several perturbed instances. LIME then fits a simple interpretable model, often a linear model or decision tree, on the perturbed instances and their corresponding predictions from the black-box model. The simple model is then evaluated for how well it approximates the black-box model for the selected instance. Finally, this simple model is used to provide explanations, in the form of importance ranking of input features, for the prediction of the black-box complex model on the selected instance.

SHAP explanations, on the other hand, is based on cooperative game theory and provides a way to assign contributions or “values” to each feature (or factor) that collectively make up a prediction. In SHAP, every explanation starts with a reference or baseline prediction. This is often the expected or average prediction of the model across the entire dataset. SHAP computes Shapley values for each feature by considering every possible combination of features and their impact on the prediction. It calculates how each feature contributes to the difference between the model’s prediction for a specific instance and the baseline prediction. Shapley values are based on the idea of fair contributions in cooperative games.

We use four state-of-the-art MHC class I predictors with different deep learning architectures and generate explanations for their outputs with both XAI techniques. The explanations for MHC class I predictors highlight important regions of the input peptide used in the output prediction which can be used for model debugging and interpretability. Interpretable explanations are key to building trust in deep learning models, in line with safety regulations such as the recently proposed EU AI act https://artificialintelligenceact.eu/the-act/ that requires explanations to help users better understand the decisions made by AI systems.

Although current XAI techniques for deep learning architectures have created a step change in providing reasons for predicted results, the question of whether the explanations themselves can be trusted has been largely ignored. Some recent studies^25–27^ have demonstrated the limitations of current XAI techniques. For instance^25^, applied three different XAI techniques on a CNN-based breast cancer classification model and found the techniques disagreed on the input features used for the predicted output and in some cases picked background regions that did not include the breast or the tumor as explanations. Literature on evaluating the reliability of XAI techniques is still in its nascency and can be broadly divided into two branches - (1) Studies that assume the availability of expert annotated ground truth, maybe in the form of bounding boxes for images, to evaluate the accuracy of explanations^28–35^ and (2) research that uses the idea of removing relevant (or important) features detected by an XAI method and verifying the accuracy degradation of the retrained models^36–43^. The first category requires human-annotated ground truth for evaluation while the second category incurs very high computational cost to verify accuracy degradation from retraining the models. Pfeifer et al.^44^ propose improving the robustness of important features by computing consensus values from multiple feature selection runs for machine learning used in biomarker discovery. This approach may have limited additional benefit in the context of XAI as most XAI techniques, like LIME and SHAP, already employ this technique, having multiple runs giving different importance rankings (for different perturbations) and combining these rankings through aggregation methods to generate a final feature importance ranking.

As our second contribution, we evaluate the quality and reliability of explanations generated by the two XAI techniques, LIME and SHAP. We do this in three different ways, (1) We check if the explanations match ground truth which in the ideal case is exact information on peptide positions involved in the binding with MHC allele. This information is, however, unavailable. Instead, we use BAlaS^45,46^ as an independent way of assessing the binding residues. BAlaS calculates the difference between the free-energy of binding of original bound complex and mutated bound complex where just one residue of ligand peptide is replaced with alanine^45,46^. (2) We check the consistency of LIME or SHAP by measuring the extent to which the explanation for a given input peptide is similar across different MHC predictors, and (3) We assess the stability of generated explanations by measuring similarity of explanations for similar input peptides with a given MHC predictor.

**Fig. 1:**
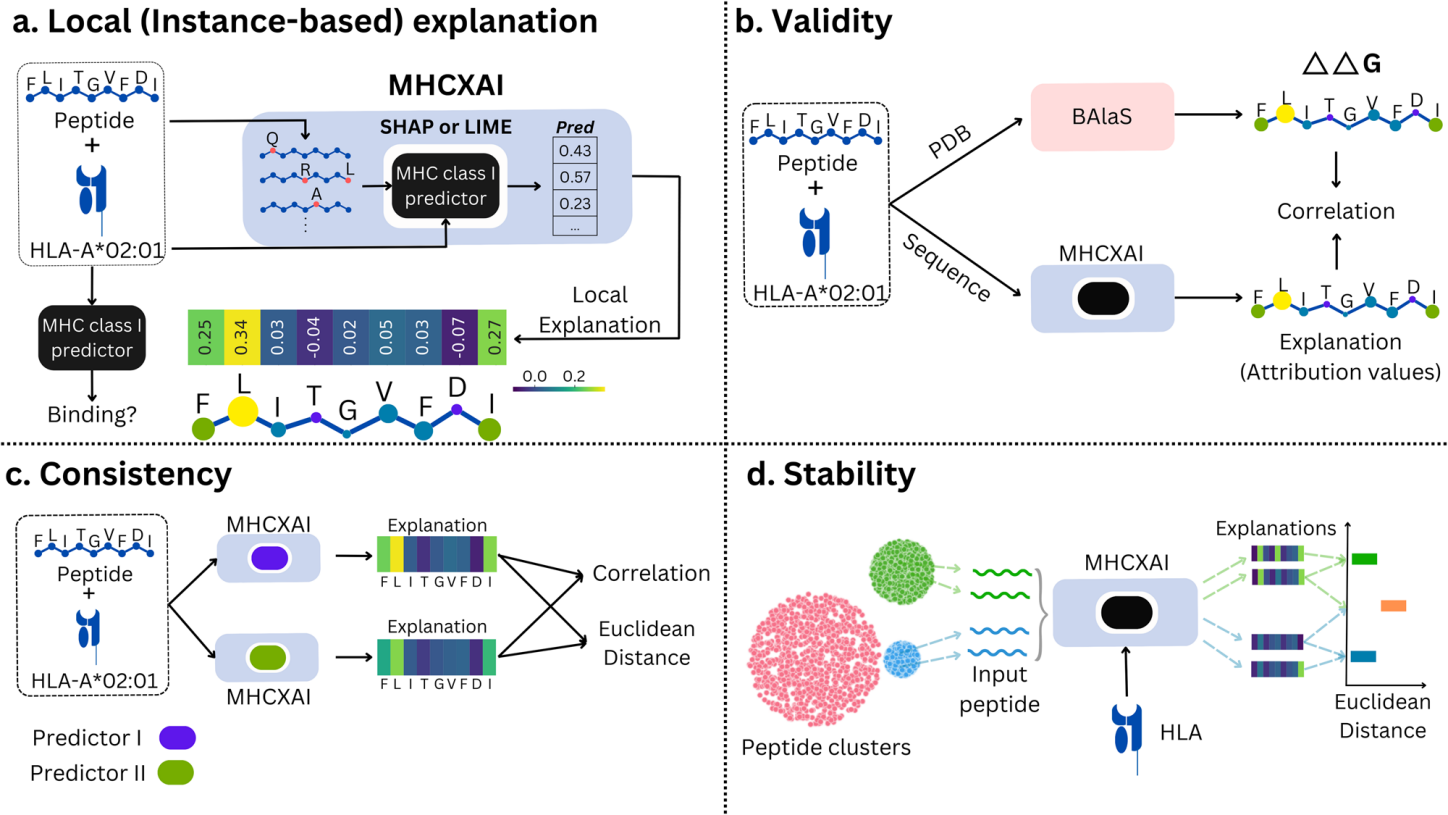
Explainable AI for MHC class I prediction. **a** MHCXAI: Framework for Generating SHAP and LIME Explanations for MHC Class I Predictors. To elucidate the prediction of an input, multiple mutated (or perturbed) copies of the input peptide are generated, and their binding probabilities are calculated using the predictor. LIME and SHAP leverage these new input-output pairs to produce explanations, i.e., attribution values for each peptide position of the original input peptide. The explanations are visualized as heatmaps. **b** The ‘Validity’ of the generated explanations is tested by comparing them against important positions highlighted by BAlaS using the PDB structure of the peptide-MHC allele bound complex. The quality of explanations is assessed through XAI metrics such as ‘Consistency’ **c** and ‘Stability’ **d**. **c** An XAI technique is deemed ‘Consistent’ if it produces similar explanations for two equally accurate predictors for a given input. We compare the two explanations using Pearson correlation coefficient and the Euclidean distance. **d** An XAI produces ‘Stable’ explanations if it generates similar explanations for similar inputs with the same prediction outcome for a given predictor. For each MHC allele, we cluster the binding peptides with GibbsCluster, comparing the Euclidean distance between the explanations for these clustered peptides within a cluster (Intracluster) and peptides across the clusters (Intercluster).

In summary, the article makes the following contributions, as shown in Fig. 1,

1. A framework, MHCXAI, that provides novel instance-based explanations for MHC class 1 predictors using XAI techniques, LIME and SHAP (Fig. 1a). As part of this contribution, we evaluate four SOTA MHC-I predictors over a large dataset, MHC-Bench, that we curate from several existing datasets.
2. Assess the validity of the explanations generated from SHAP and LIME by comparing against BAlaS-based ground truth (Fig. 1b).
3. Assess quality of the explanations generated from two explainable AI techniques, shown in Fig. 1c, d, with respect to consistency and stability of explanations.

We also provide additional analysis of explanations in Supplementary Material (see Supplementary Note 1 which summarizes all the contributions in the supplementary material).

## Results

### MHC-Bench

The MHC-Bench dataset consists of 2,232,937 peptides of length 9 and 115 MHC alleles. All the MHC alleles in this dataset are human MHC molecules (i.e. Human Leukocyte Antigens or HLA). Out of the 115 MHC alleles, about half are HLA-B, a third are HLA-A and remaining HLA-C. The MHC-Bench dataset contains 3,464,013 peptide-MHC pairs previously unseen by predictors during training. It is worth noting that the peptides by themselves may have been seen in the training data paired with a different HLA allele. The peptide overlap between training data for investigated predictors and MHC-Bench is shown in Table 1. A description of the construction and composition of the dataset is presented in Methods section.

**Table 1:** Overlap between training peptides of MHC class I predictors and MHC-Bench dataset peptides

MHC class I predictor	Number of training peptides	Number of overlapping peptides	Overlap % with MHC-Bench
MHCflurry	190,095	95,743	4.29%
MHCfovea	108,092	50,072	2.24%
NetMHCpan	31,212	1,149	0.05%
TransPHLA	1,184,191	289,605	12.97%

### MHC class I predictors

We evaluated the performance of four MHC class I predictors on the MHC-Bench dataset – MHCflurry, MHCfovea, NetMHCpan and TransPHLA. The choice of the four predictors was guided by their popularity and performance reported in the literature^1,2,4,47^. MHCflurry–2.0^47^ is an ensemble predictor of 10 neural networks for predicting presentation of a peptide. It supports 14,993 MHC class I alleles. MHCflurry provides three scores, namely – Binding Affinity (BA), Processing score and Presentation Score (PS). PS is produced by a logistic regression model combining the binding affinity and processing scores. Processing score captures the antigen probability which combined with binding affinity substantially improves the performance of the predictor^47^.

NetMHCpan–4.1^1^, an ensemble of 50 neural network models, produces Elution Ligand (EL) and Binding Affinity (BA) score and we refer to these modes as NetMHCpan-EL and NetMHCpan-BA respectively. It utilizes NNAlign_MA^48^, an artificial neural network that predict both BA and EL scores. For both modes, peptides with rank 2% or less are considered as binders and 0.5% or less as strong binders. It supports 11,000 MHC class I alleles.

MHCfovea^2^, is an ensemble of multiple CNN models that takes MHC allele sequence and peptide sequence as input to predict binding probability. In MHCfovea, ScoreCAM^2,49^ is applied to identify important positions and corresponding residues in input peptides and MHC allele sequence. It provides the motifs for the first and last 4 positions of the peptide for each allele along with the motif for MHC sequence.

TransPHLA^4^, is a transformer architecture that predicts binding probability for an input peptide and MHC allele sequence. Using the attention scores, important residues for each position of a 9–*mer* can be obtain to generate a peptide motif for a given allele.

### Benchmarking performance

**Fig. 2:**
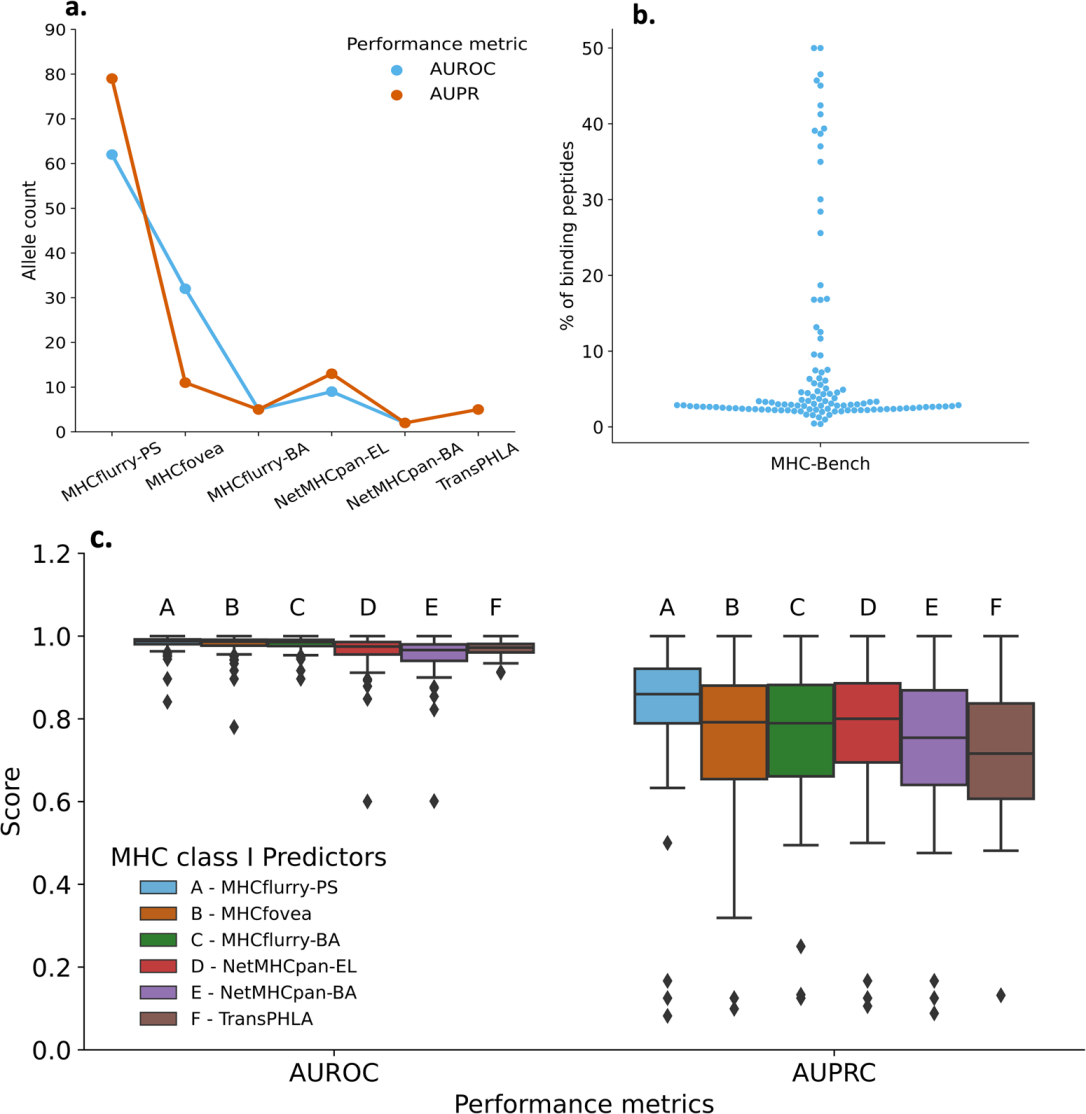
Benchmarking the performance of investigated MHC class I predictors. **a** The number of alleles for which predictors exhibited the highest performances based on AUROC and AUPRC scores. **b** The percentage of binders in the MHC-Bench dataset per allele. Each dot represents a measurement for one allele. **c** Distribution of AUROC and AUPRC scores for the MHC class I predictors.

We use Area Under ROC (AUROC) and Area Under Precision-Recall Curve (AUPRC) as benchmark or performance metrics. Since peptide binding is MHC allele specific, we calculated the scores for each allele separately. The benchmark metrics for each allele are reported in Supplementary Data 1, 2. The average benchmark metrics for the MHC class I predictors are reported in Table 2. We find that all four predictors are comparable in their average performances across all alleles, as seen in Fig. 2c. While the differences between the scores among predictors is minimal, we see that for most alleles, performance of MHCflurry-PS is marginally higher. Figure 2a displays number of times (or number of alleles) the predictor was top performer based on AUROC and AUPRC score. We provide additional benchmarking analysis of the predictors in the Supplementary material (See Supplemental Fig. 1 and Supplementary Note 2) which indicates that the performance of the predictors are comparable across various metrics. The predictors achieve a higher performance on the AUROC metric, 0.95–0.98, as opposed to AUPRC where it is in the range of 0.75–0.86. For most alleles, there are fewer binding peptide-MHC pairs in comparison to non-binding peptide-MHC pairs. This is evident in the distribution plot in Fig. 2b where for most of the alleles, only 1–10% of the peptides are binding. The %binding peptide-MHC pairs per allele is provided in Supplementary Data 3). In imbalanced scenarios with few positive labels, the AUROC metric can be “overly optimistic”^50^. In contrast, AUPRC is relatively unaffected by this imbalance in the dataset owing to its focus on true positives (as true negatives are not into consideration). These two metrics were selected because they are used for benchmarking in the original paper describing the predictors. Recent work by Carrington et al.^51^ provides a useful discussion on use of AUROC and AUPRC metrics while also introducing a new metric called DeepROC.

**Table 2:** Performance of MHC class I predictors (mean scores)

Predictor	AUROC	AUPRC
MHCflurry-PS	0.983 ± 0.019	0.858 ± 0.141
MHCfovea	0.979 ± 0.025	0.795 ± 0.169
MHCflurry-BA	0.980 ± 0.017	0.794 ± 0.156
NetMHCpan-EL	0.964 ± 0.043	0.806 ± 0.158
NetMHCpan-BA	0.955 ± 0.046	0.766 ± 0.167
TransPHLA	0.970 ± 0.0173	0.752 ± 0.148

### Explanations For MHC class I predictors

Explanations can be classified as either “Global” or “Local”. Global explanations for a predictor are distribution of input feature values across “all” inputs in the dataset. This offers a consolidated perspective on how the model utilizes input features for a specific output label. In contrast, local (or instance-based) explanations, focus on a single input - output instance. Local explanations typically show attribution of input features used for the prediction outcome. In the context of MHC class I predictors, binding motifs are examples of global explanations while a vector of attribution values for individual peptide positions forms a local explanation. It is worth noting that our work focuses on post-hoc explanations, i.e., explanations for existing models that have been previously trained. Post-hoc explanations are widely applicable as they can be used over models whose internal structure is not known or is too complex to interpret. Existing MHC class I predictors, like MHCfovea, focus on global explanations by generating binding motifs for MHC alleles. There is limited work on local instance-based explanations for this problem. In the next two sections, we motivate the need for local explanations for MHC class I predictors and discuss the additional information it can provide over global explanations.

### Local instance-based explanations using LIME and SHAP

An explanation for a 9–mer peptide in our instance-based approach is represented as a length-9 vector of attribution values, generated through LIME or SHAP. Each position’s attribution value can be positive or negative, with a positive (or negative) value indicating that the residue at that position contributes positively (or negatively) to the prediction. Our MHCXAI framework facilitates the generation of explanations for any MHC class I predictor by simply substituting the predictor module while keeping the LIME and SHAP modules unchanged. Using the MHCXAI framework, we successfully generated LIME and SHAP explanation vectors for input peptides from the MHC-Bench dataset across all examined predictors.

In this study, our main emphasis is on providing explanations for input peptides rather than allele sequences, given that not all predictors can process allele input in the form of an amino acid sequence. For instance, NetMHCpan and MHCflurry accept allele inputs as HLA-A02:01 and HLA-A0201, respectively. However, it is important to highlight that our framework has the capability to generate explanations for allele sequences in addition to peptides. This feature can be particularly beneficial for predictors like TransPHLA, which accepts the allele as an input sequence. An illustration of an allele explanation is presented in Supplementary Fig. S2 of Supplementary Note 3.

**Fig. 3:**
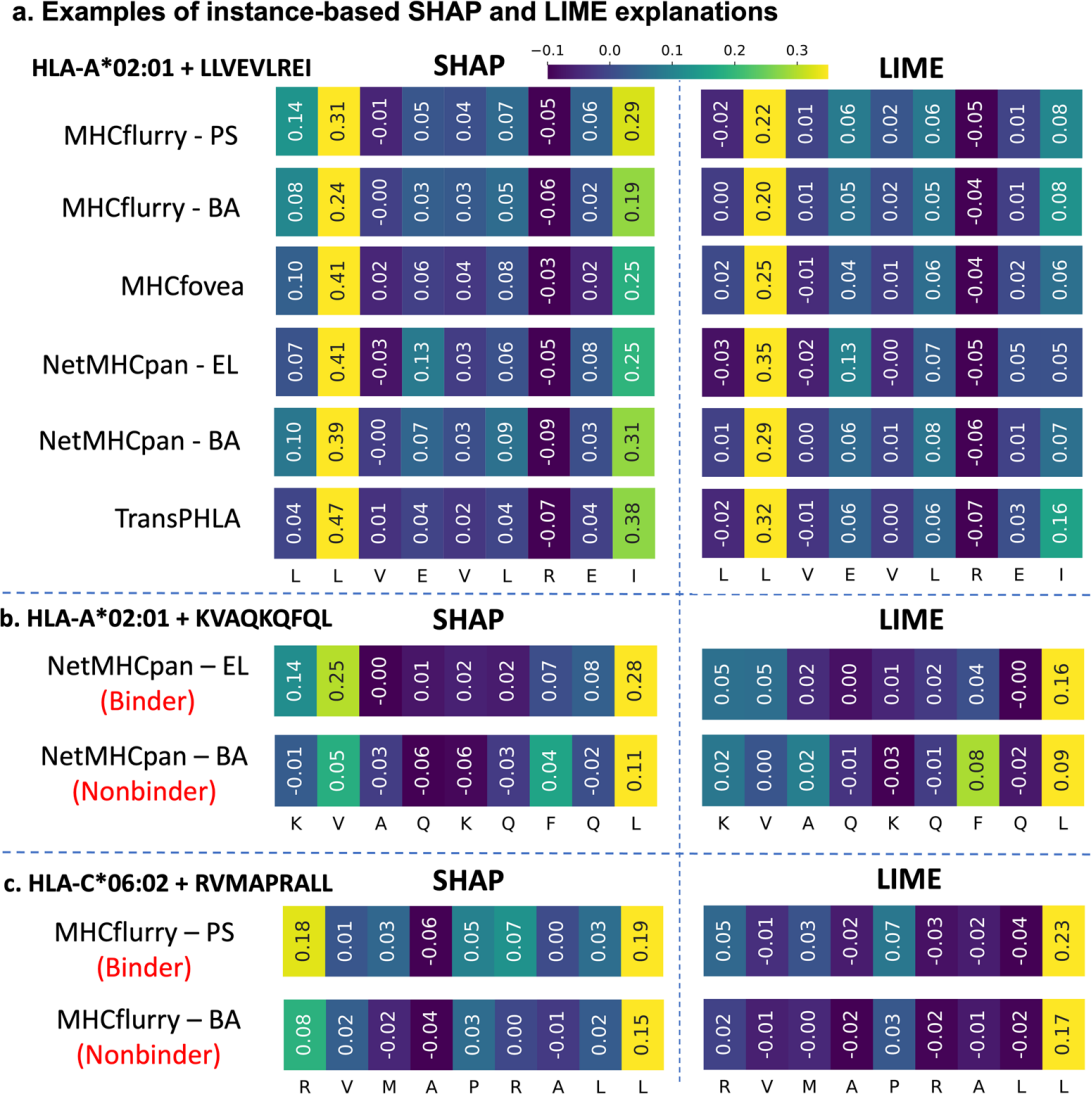
Instance-based (local) explanations for the Investigated MHC Class I Predictors. **a** Examples of SHAP and LIME explanations for all investigated MHC class I predictors for the LLVEVLREI--HLA-A*02:01 pair. To visualize the explanations, the attribution values of positions are used to create heatmaps. For each explanation, a lighter color indicates a positive contribution, while a darker color indicates a smaller or negative contribution to the positive class. SHAP explanations for all the predictors highlight peptide positions P2 and P9 as the most important for binding, while LIME explanations highlight only peptide position P2 as the most important. **b** LIME and SHAP explanations for NetMHCpan-EL and -BA for the peptide KVAQKQFQL binding to HLA-A*02:01. NetMHCpan-EL classifies KVAQKQFQL correctly, but NetMHCpan-BA does not. SHAP captures these differences in performance and produces different explanations for the two predictor modes. **c** LIME and SHAP explanations for MHCflurry-PS and -BA for the peptide RVMAPRALL binding to HLA-C^*^06: 02. MHCflurry-PS classifies RVMAPRALL correctly, but MHCflurry-BA does not. Similar to the example in **b**, SHAP captures these differences in performance and produces different explanations for the two predictor modes. In both NetMHCpan and MHCflurry examples, LIME explanations are unable to indicate positions leading to the difference in prediction outcome.

Figure 3a illustrates LIME and SHAP explanations generated for all examined predictors for a specific peptide-MHC allele pair (LLVEVLREI–HLA-A*02:01). To visualize the explanations, heatmaps are constructed using the attribution values, with lighter colors indicating positive contributions and darker colors indicating little or negative contributions to the binding class. The peptide LLVEVLREI is a binding peptide for the HLA-A*02:01 allele, correctly classified by all MHC class I predictors. However, it is worth noting that the explanations from SHAP and LIME exhibit slight differences. For example, in Fig. 3a, both LIME and SHAP attribute high importance to peptide position P2, but SHAP also recognizes peptide position P9 as significant. P9 is typically considered important for binding based on existing literature^52,53^.

In Fig. 3b, the peptide KVAQKQFQL binds to the MHC allele HLA-A*02:01. However, within NetMHCpan, NetMHCpan-EL correctly predicts it as a binder, while NetMHCpan-BA classifies it as a non-binder. SHAP and LIME explanations were generated for both modes, revealing that SHAP can identify the features (or peptide positions) responsible for the predictions made by NetMHCpan-EL and -BA. For instance, at peptide position P1, the amino acid “K” positively contributes to the prediction outcome in NetMHCpan-EL, whereas it negatively contributes in NetMHCpan-BA. LIME, on the other hand, produces similar attribution values for both predictors and is unable to highlight the cause for the difference in prediction between the two NetMHCpan modes.

In Fig. 3c, the peptide RVMAPRALL is a binder to the MHC allele HLA-C*06:02, classified as a binder by MHCflurry-PS but not by MHCflurry-BA. SHAP and LIME were employed to explore the difference in predictions. SHAP identified that for MHCflurry-PS, peptide positions P1 and P9 play an important role. For MHCflurry-BA, while peptide position P9 is crucial, P1 is not deemed important (refer to the heatmaps in Fig. 3c). This distinction between the two explanations helps identify and understand the reasons for different predictions.

These examples demonstrate that XAI techniques can generate explanations for MHC class I predictors. However, explanations produced by distinct XAI techniques for the same predictor may not align. Consequently, we evaluate the validity and quality of LIME and SHAP explanations against XAI metrics.

The average time required to generate an explanation for a single instance is influenced more by the choice of the predictor than the XAI technique itself. For example, generating an explanation (either LIME or SHAP) for MHCfovea takes twice as long as generating a corresponding explanation for MHCflurry.

### Contrasting local explanations against binding motifs

As stated earlier, global explanations for MHC class I predictors manifest as binding motifs. Determining the binding preference for an MHC allele involves examining the most frequently occurring amino acids at anchor positions, specifically positions 2 and 9 in a 9–mer peptide, which are the primary sites responsible for binding to an MHC molecule^52,53^. This can be extended to other peptide positions, forming a binding motif for an MHC allele.

Biological binding motifs for MHC alleles are generated using experimentally validated strong binders^54^. With recent MHC class I predictors, binding motifs are derived from peptides predicted as strong binders for a particular allele. These peptides serve as the basis for generating position-specific scoring matrices (PSSM), which are then visually represented as binding motifs. This approach is applied to generate binding motifs for MHCflurry, NetMHCpan, and MHCfovea. By comparing these predictor-generated motifs against the biological motif, it becomes possible to assess whether the predictor has effectively learned the correct binding patterns for a given allele. In this study, we utilize binding motifs from the MHC Motif Atlas database^55^.

Global explanations may overlook deviations observed in specific inputs. Consider a binding peptide that diverges from the typical biological binding motif pattern. In cases where a predictor correctly classifies this peptide as a binder, it becomes valuable to examine the specific features used by the predictor for this classification. Understanding the input features employed by the predictor for a particular peptide requires a local explanation rather than a binding motif. Specialized patterns like these are difficult to infer with a binding motif.

**Fig. 4:**
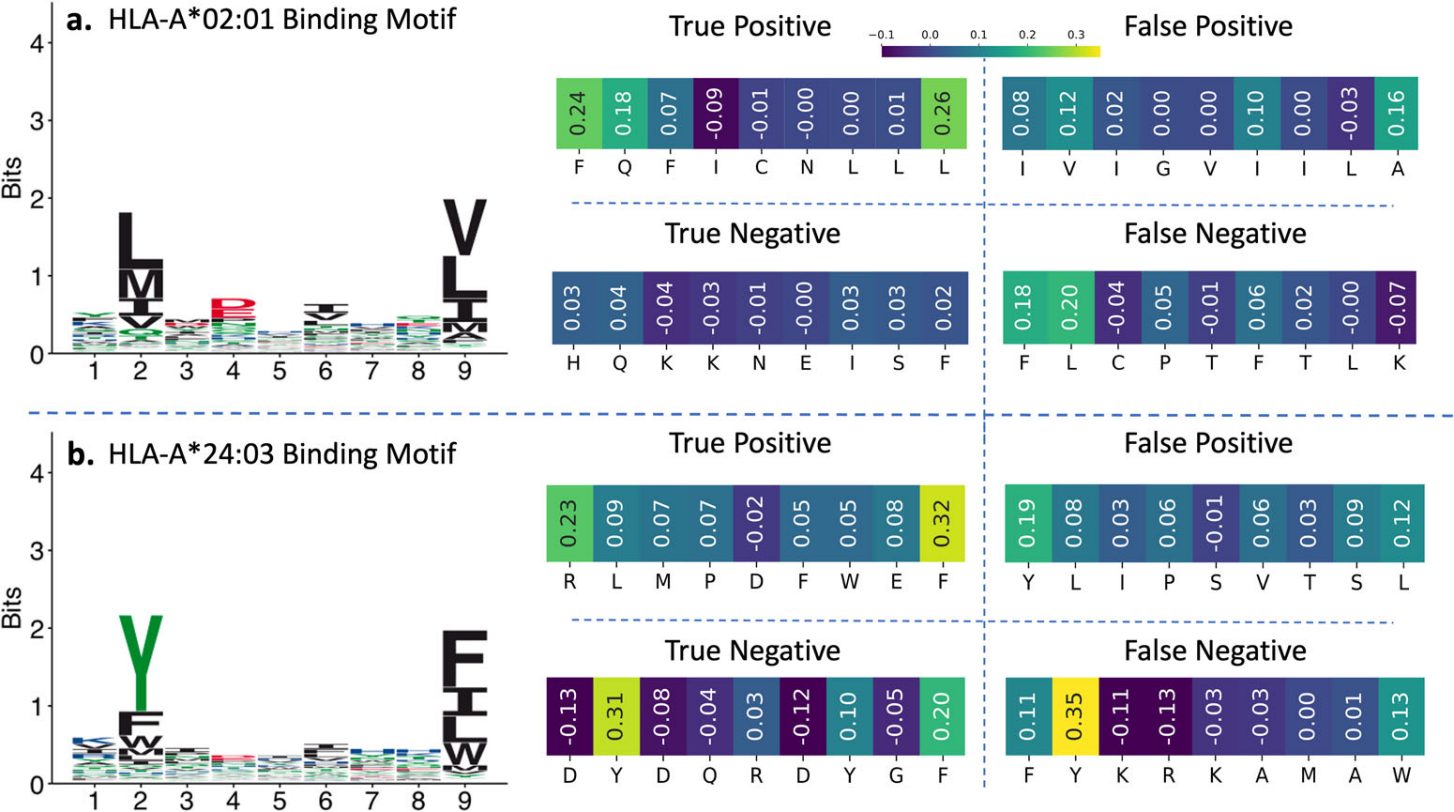
Instance-based (local) explanations for correctly and incorrectly classified peptides along with corresponding MHC allele binding motifs. For **a** and **b**, biological binding motifs for HLA-A*02:01 and HLA-A*24:03 are obtained from the MHC Motif Atlas^55^. For each MHC allele, there are four heatmaps, which are SHAP explanations generated for true positive, true negative, false positive, and false negative peptides predicted using MHCflurry-PS. The peptides in both **a** and **b** defy the reasoning for binding based on biological motifs. However, the SHAP explanations are able to highlight the cause behind unexpected outcomes. A lighter color in the explanation heatmap indicates a positive contribution, while a darker color indicates a smaller or negative contribution to the positive class.

We illustrate the necessity for local explanations to capture specialized patterns that deviate from biological binding motifs with specific examples. In Fig. 4a, the motif for HLA-A*02:01 suggests a preference for amino acids “L”, “I”, and, “M” at anchor position P2, binding to the super hydrophobic “B” pocket of the MHC molecule. However, despite the unfavorable nature of water-soluble Glutamine (“Q”) for such a pocket, solved peptide-bound HLA molecule structures indicate that many peptides with “Q” do bind strongly to HLA-A*02:01^56^. An example is the peptide FQFICNLLL (see Fig. 4a), correctly classified as a binder by the MHCflurry-PS predictor. We generated a local explanation for this peptide using SHAP (see Fig. 4a). The highest attribution values were assigned to peptide positions P1, P2, and P9. While the high importance of peptide positions P2 and P9 aligns with their roles as anchor positions, the elevated attribution value for peptide position P1 is rationalized by its crucial role in stabilizing the bound structure, as observed in refs. ^53,56,57^. It is worth noting that the amino acid “Q” in position P2 does not appear in the biological binding motif (global explanation) prominently which fails to capture the specialized pattern in this instance.

In Fig. 4a, the true negative instance HQKKNEISF also contains the amino acid “Q” in position P2, similar to the true positive instance discussed. However, in this case the local explanation shows low attribution values for all other peptide positions. This indicates lack of strong binding signal from those positions, explaining the negative classification.

Figure 4b is another example of explanations generated for true positive, true negative, false positive and false negative predictions made by MHCflurry-PS for HLA-A*24:03. In this example, peptide conforming to binding motif is correctly classified as non-binder whereas peptide not conforming to binding motif is correctly classified as binder.

In summary, instance-based explanations are particularly useful in explaining scenarios where binder peptides do not conform to motifs, misclassifications, and understanding a peptide specific pattern used for prediction. Additionally, we show that global explanations can be created using instance-based SHAP and LIME explanations in Supplementary Fig. S3 (in Supplementary Note 4) which can be useful for quick comparison across predictors.

### ΔΔ*G* for validity of explanation

To achieve trust in a predictor, the generated explanations for the predictions must be reliable. Assessing the reliability of explanations involves comparing them to ground truth about the input-output relationship, as suggested by prior work such as^58–60^. The ground truth in our case are the residues in the peptide that genuinely contribute to the binding. The contribution of residues (‘hotspots’) can be estimated experimentally using Alanine-scanning mutagenesis^61^, a resource intensive^45^ technique. Computationally, this can be achieved using BAlaS^45,46^ which calculates the difference between the free-energy of binding of original bound complex and mutated bound complex where just one residue of ligand peptide is replaced with alanine. This difference in free-energy of binding is indicated as ΔΔ*G* and ΔΔ*G* ≥ 4.184 kJ/mol is considered ‘hot’ or important residue for binding^46^. ΔΔ*G* ≤ − 4.184 kJ/mol indicates alanine enhances binding relative to the original residue^46^. Any value between denotes neutral substitution^46^. As it is difficult to obtain ground truth for all peptides, we use this ΔΔ*G* as an independent way of highlighting important residues in the peptide.

First, we compile all the available PDB structures featuring bound peptide-MHC allele complexes as documented in the MHC Motif Atlas^55^. Subsequently, we refine the list to encompass bound peptides with a length of 9 and narrow down the selection to structures that were consistently classified as binders by all examined MHC class I predictors. The resulting list comprises 250 PDB structures, encompassing 40 distinct MHC alleles (as listed in Supplementary Data 5).

We compared the ΔΔ*G* for these 250 peptide-MHC pairs with the LIME and SHAP explanations generated for all the predictors. The LIME and SHAP values can be positive or negative, similar to ΔΔ*G*, which indicates residue contribution to the prediction. For each of these 250 peptide-MHC pair we calculated Pearson correlation coefficients between LIME/SHAP explanations and ΔΔ*G*, for each of the investigated predictors.

**Fig. 5:**
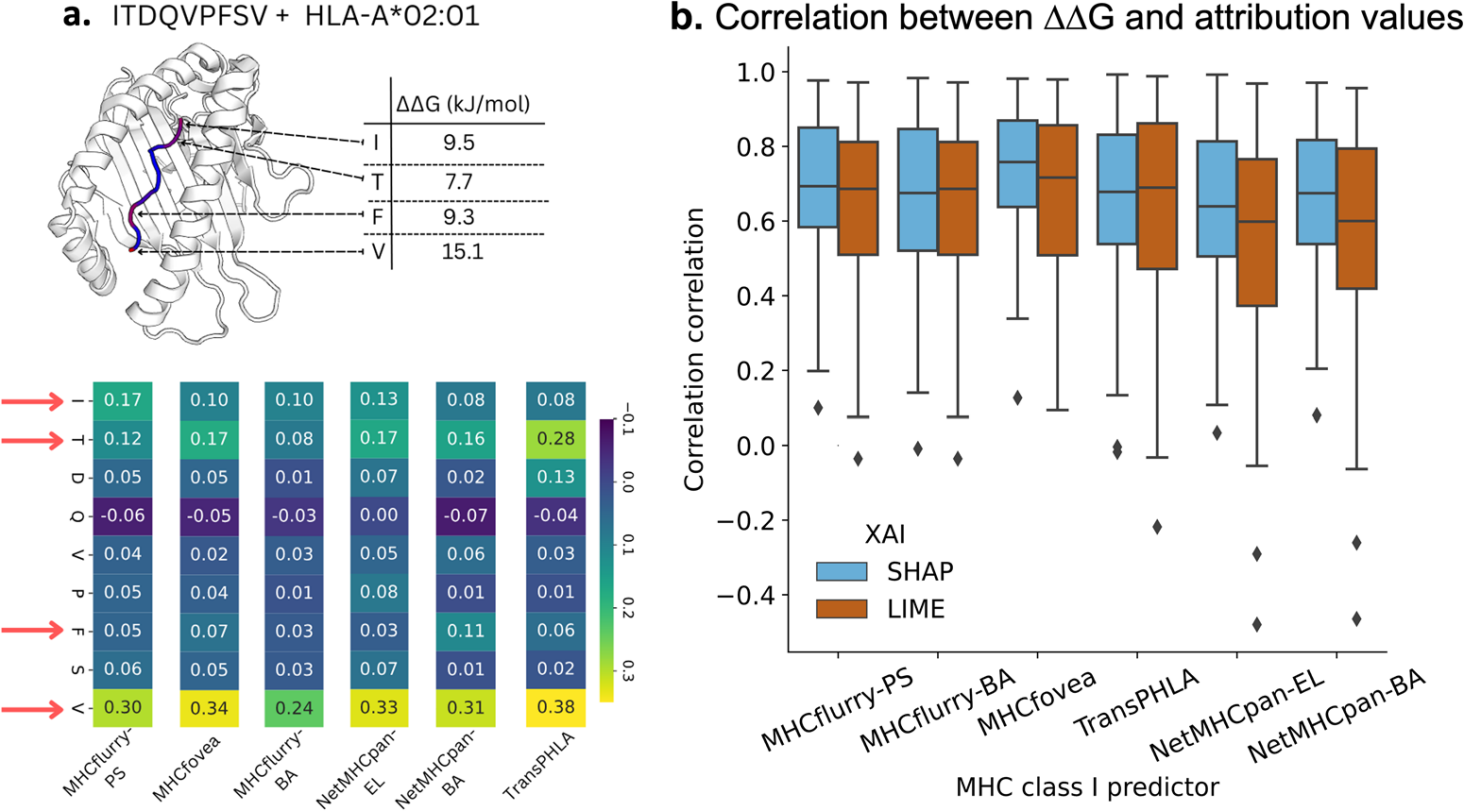
Validation of the explanations. It is done by comparing the attribution values to the difference in free-energy of binding between wild-type protein-protein interaction and mutated protein-protein interaction, known as ΔΔ*G*. **a** For the ITDQVPFSV--HLA-A*02:01 complex, BAlaS highlights that peptide positions P1, P2, P7, and P9 are crucial for binding. Replacing the residues at these positions with alanine leads to an increase in ΔΔ*G*, indicating instability. This peptide is correctly classified by all the investigated MHC class I predictors, and SHAP explanations are generated for each of them. The explanations mostly match the ground truth, as P1, P2, P7, and P9 (indicated by red arrows) are rightly highlighted as the factors influencing the prediction. **b** ΔΔ*G* was calculated for each peptide position in 250 PDB structures containing peptide-MHC allele bound complexes that were correctly classified by all the investigated predictors. The SHAP and LIME explanations correlated positively for most complexes, indicating that the explanations mostly align with the ground truth and can be trusted. The correlation coefficient values are reported in Supplementary Data 4. A lighter color in the explanation heatmap indicates a positive contribution, while a darker color indicates a smaller or negative contribution to the positive class.

In Fig. 5a, consider the instance of the peptide ITDQVPFSV bound to HLA-A*02:01, which is correctly classified. BAlaS identifies peptide positions P1, P2, P7, and P9 as ‘hot’ residues (with ΔΔ*G* ≥ 4.184 kJ/mol), which are highlighted in red within the peptide-MHC complex. The SHAP explanations, feature red arrows pointing to the positions identified as important by BAlaS. Generally, the models consistently prioritize these positions when making predictions. However, despite having a high ΔΔ*G*, peptide position P7 is not deemed important by most of the predictors. This suggests that the information from the other three residues is sufficient for the predictors to infer the classification outcome. The distribution of correlation coefficients between SHAP-ΔΔ*G* and LIME-ΔΔ*G* (depicted in Fig. 5b) indicates a positive correlation between the explanations and the important positions identified by BAlaS. Overall, it is observed that SHAP explanations exhibit a closer correlation compared to LIME explanations.

The observed variance in the distribution of correlation coefficients is not surprising, given that BAlaS ΔΔ*G* serves as only an approximation of the actual positions involved in binding, and the approach is subject to certain limitations. Notably, the accuracy of the ΔΔ*G* calculation is influenced by the resolution of the PDB structure (refer to Supplementary Fig. S4 and Supplementary Note 5). To address this, we selectively choose PDB structures with the highest resolution when multiple structures are available. Additionally, since ΔΔ*G* is computed by substituting a residue with alanine, it is challenging to ascertain the contribution of alanine, if present (refer to Supplementary Fig. S5).

### Consistency

Consistency refers to similarity in the explanations produced for two similarly performing predictors on a given input. We assess consistency of an XAI technique by comparing explanations for a given peptide between two similarly performing MHC class I predictors (Fig. 1c).

**Fig. 6:**
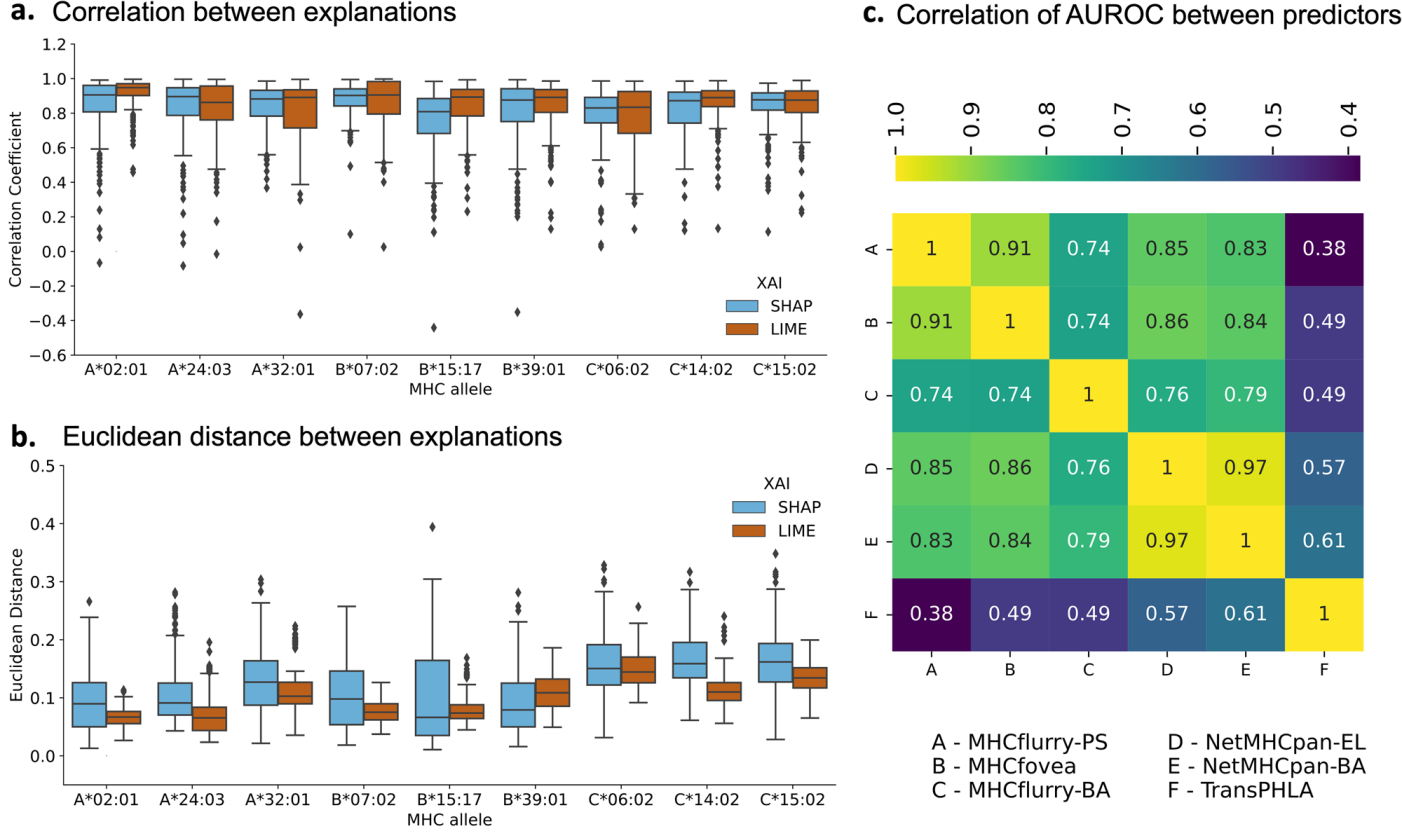
Testing the consistency of the LIME and SHAP explanations. **a** For an input peptide, explanations were generated for MHCflurry-PS and MHCfovea using SHAP and LIME. Pearson correlation coefficients were calculated between these explanations, and the process was repeated for 200 input peptides for each of the alleles presented in the plot. The distribution of Pearson correlation coefficients is closer to one, indicating high similarity between the two explanations for the two predictors on the same input. The correlation coefficient values are reported in Supplementary Data 6. **b** In addition to the Pearson correlation coefficient, Euclidean distances were calculated between two explanations for two predictors on the same input. For Euclidean distance, values closer to zero indicate high similarity and high consistency. **c** Correlation heatmap for AUROC scores between investigated MHC class I predictors. MHCflurry-PS and MHCfovea are highly correlated in their performances.

To select two predictors with comparable performance, we choose the top two predictors from our results in Section 2 (see Fig. 2a), namely MHCflurry-PS and MHCfovea. Additionally, the AUROC scores for these two predictors exhibit a high correlation, as indicated in Fig. 6c, demonstrating substantial similarity in their performance.

We selected 9 alleles (3 each from HLA-A, B and C) and for each allele, we randomly selected 200 peptides from our MHC-Bench dataset to generate local explanations, independently using each of SHAP and LIME, for MHCfovea and MHCflurry-PS. For both LIME and SHAP, to compare the similarity between the explanations from the two predictors, we computed Pearson correlation and Euclidean distance.

In Fig. 6a, the distribution of Pearson correlations between explanations generated individually for MHCflurry-PS and MHCfovea using LIME and SHAP is presented for all nine alleles. Overall, the majority of the SHAP and LIME explanations exhibited high correlation. In Fig. 6b, the distribution of Euclidean distances between the explanations of the two predictors is presented. Explanations that are similar will have a Euclidean distance closer to zero. It is noteworthy that the Euclidean distance distribution for LIME has a narrow range and tends to be closer to zero compared to SHAP. This observation suggests that LIME produces more consistent explanations compared to SHAP.

We also created a baseline distance between explanations for the two predictors using the following procedure. First, we generated 100 random explanations for each original MHCfovea explanation by randomly permuting the attribution values. Next, we calculated the distance between each of these 100 random explanations and the original MHCflurry-PS explanation. The baseline distance was then computed by averaging these 100 distances. This process was repeated for all 200 peptides chosen per allele. Consequently, we obtained 200 Euclidean distances between the original MHCflurry-PS and MHCfovea explanations, along with their corresponding baseline distances. We compared these two distributions for each allele. We confirmed that the two distributions for both SHAP and LIME were statistically different using Kruskal-Wallis test at 5% significance level. The p-value, H-statistics and effect size are reported in Supplementary Tables 1 and 2 for SHAP and LIME respectively. It is also worth noting that the Euclidean distance for both LIME and SHAP explanations were smaller than the corresponding average baseline Euclidean distance for nearly all the input peptides (99% input peptides).

We confirmed that the two distributions for both SHAP and LIME were statistically different using Kruskal-Wallis test at 5% significance level. The p-value, H-statistics, and effect size are reported in Supplementary Tables 1 and 2 for SHAP and LIME, respectively. Additionally, it is noteworthy that the Euclidean distances for both LIME and SHAP explanations were smaller than the corresponding average baseline Euclidean distances for nearly all input peptides (99% of input peptides).

### Stability

Stability of an explanation technique refers to the extent to which explanations for similar inputs (with same output) over a given predictor are close. We use the MHCflurry-PS predictor to asses stability of the LIME and SHAP techniques, independently. To identify input peptides that are similar, we perform clustering over a subset of peptide sequences for HLA-A*02:01. Using GibbsCluster-2.0^62,63^, we cluster the peptides into 1–10 clusters. The number of clusters that yields highest average Kullback–Leibler Distance (KLD) is considered to be the optimum number of clusters. We found choosing 10 clusters has the highest KLD with cluster size ranging between 700–1000 peptides. The plot showing KLD distribution and cluster motifs generated from GibbCluster is provided in Supplementary Fig. S6. Peptides within a cluster are considered similar.

**Fig. 7:**
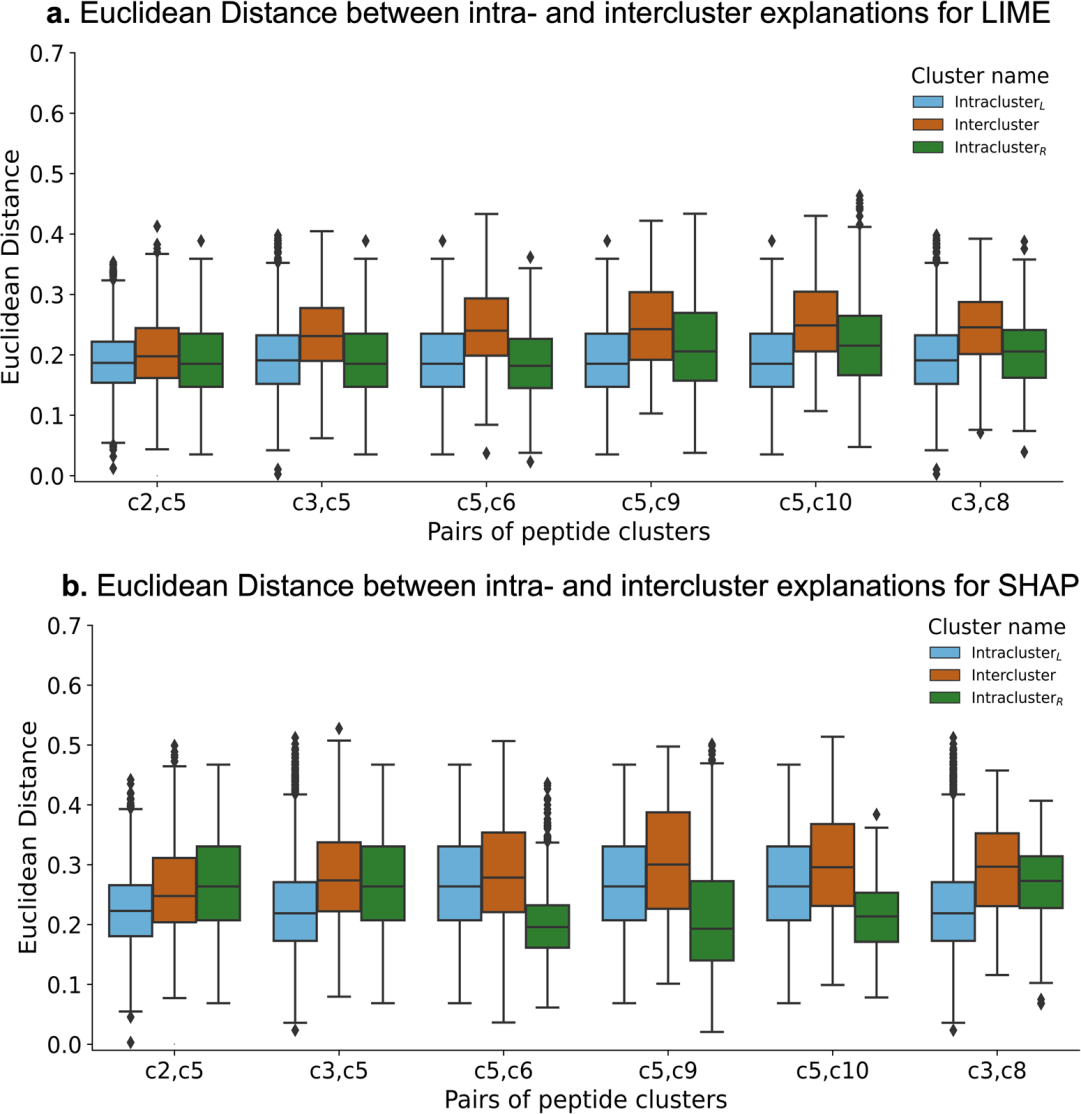
Testing the stability of the LIME and SHAP explanations. **a** Euclidean distance distribution for the top six cluster pairs using LIME. For each pair, there are three distributions - Intracluster_*L*_, Intercluster, and Intracluster_*R*_. For any two pairs (e.g., c2, c5), the intracluster explanation distance distribution for the left cluster (c2) and right cluster (c5) are Intracluster_*L*_ and Intracluster_*R*_, while Intercluster is the distribution of explanation distances between the two clusters. **b** Euclidean distance distribution for the top six cluster pairs using SHAP. For each pair, there are three distributions - Intracluster_*L*_, Intercluster, and Intracluster_*R*_. For any two pairs (e.g., c2, c5), the intracluster explanation distance distribution for the left cluster (c2) and right cluster (c5) are Intracluster_*L*_ and Intracluster_*R*_, while Intercluster is the distribution of explanation distances between the two clusters.

From each of these clusters, we sampled 100 peptides that are binders and generated explanations for these peptides. We calculated the Euclidean distance between all pairs of peptides within each cluster, and this is referred to as the intracluster distance distribution. As a comparison, we also computed the distance between explanations for peptides from different clusters, referred to as Intercluster distance. We show results for the top six most unrelated cluster pairs – (c2, c5), (c3, c5), (c3, c8), (c5, c6), (c5, c9), (c5, c10), based on the similarity of their position-specific scoring matrix, in Fig. 7.

For each cluster pair in Fig. 7, we have three distributions: Intracluster_*L*_, Intercluster, and Intracluster_*R*_. Consider the pair (c2, c5), where Intracluster_*L*_ represents the intracluster Euclidean distance distribution for cluster c2 (or the left cluster), Intercluster is the intercluster Euclidean distance distribution between c2 and c5, and Intracluster_*R*_ is the intracluster Euclidean distance distribution for cluster c5 (or the right cluster). The notation of left-right for Intracluster is arbitrary. It is worth noting that intracluster distances are lower than the intercluster distances, indicating that LIME and SHAP explanations for peptides within the same cluster are more similar, suggesting stability of explanations. We confirmed that the differences between intracluster and intercluster distance distributions in Fig. 7 are statistically significant (Kruskal–Wallis test). The *p*-value, H-statistics, and effect size are reported in Supplementary Table 3.

## Discussion

In recent times, explainable AI techniques have garnered significant attention as a means of fostering trust in deep learning models by providing interpretability of model decisions. Initiatives like the DARPA 2017 XAI research program and the EU AI Act aim to increase the trustworthiness of AI models by mandating explanations that enable users to better comprehend the decisions made by AI systems. Transparency in AI models is particularly crucial in high-stakes scenarios, such as biomedical research. In an effort to bridge the gap between the growing importance of XAI and its application in the biomedical space, we have explored the applicability of XAI to MHC class I prediction.

In this study, we employed two popular explainable AI (XAI) techniques, namely SHAP and LIME, to generate explanations for four top-performing MHC class I predictors—MHCflurry, MHCfovea, NetMHCpan, and TransPHLA. We highlight the necessity of instance-based or local explanations in conjunction with global explanations for a comprehensive understanding of the predictors. The validity of the explanations was assessed by comparing them to independently derived important peptide positions obtained from BAlaS of PDB structures of peptide-MHC allele bound complexes. Additionally, we evaluated the quality of explanations using XAI metrics, namely consistency and stability. Overall, our findings indicate that both LIME and SHAP produce valid explanations that are consistent and stable. Moreover, the LIME and SHAP are largely in agreement with each other (see Supplementary Note 8 and Supplementary Fig. S9). While LIME explanations exhibit greater stability and consistency across predictors and similar peptides than SHAP, SHAP explanations are more accurate in assessing importance of amino acid and positions within a given peptide towards the peptide-MHC binding outcome.

The provided explanations and their evaluation will aid in interpreting the output of MHC class I predictors, building trust in their decisions. The contributions in this article have the potential for generalization and can be readily adapted to interpret and instill trust in other deep learning models over biological sequences.

## Methods

### Dataset-MHC-Bench

The ‘MHC-Bench’ dataset is curated by combining multiple existing datasets, namely – Therapeutics Data Commons (TDC)^64,65^, the external and independent dataset from TransPHLA^4^, monoallelic benchmark dataset from MHCflurry^47^ and benchmark dataset from NetMHCpan^1^. We constrained our dataset to peptides with a length of 9. Peptide-MHC allele combinations present in the training data for NetMHCpan–4.1, MHCflurry–2.0, MHCfovea, and TransPHLA were removed to ensure fairness in the evaluation of the different MHC class I predictors. Additionally, we excluded peptide-MHC allele combinations with conflicting labels and eliminated MHC alleles where only one class is represented (i.e., either all peptides are binders or non-binders). The final benchmark dataset, named MHC-Bench, comprised 115 MHC alleles and 2,232,937 peptides, forming 3,464,013 peptide-MHC allele combinations. All the MHC alleles belonged to the Human Leukocyte Antigens (HLA).

### Explainable AI methods

For this study, we utilized SHapley Additive exPlanations (SHAP)^24^ and LIME^10^ to generate explanations. We developed a framework named MHCXAI for applying SHAP or LIME to MHC class I predictors.

SHAP, proposed by Lundberg and Lee^24^, this model-agnostic approach employs Shapley values, a concept from game theory, to determine the contribution of each feature to the model’s output. The method involves starting from one random position mutation, followed by mutating other positions until the correct model classification is achieved. This process is repeated multiple times with random perturbations to obtain the importance of each position, represented as Shapley values. To reduce the variance in SHAP values, we set the number of times the model evaluations to be 25,000 (see convergence study in Supplementary Note 6 and Supplementary Fig. S7). SHAP requires a training dataset for each model to generate the background distribution for sampling. However, the training data for many predictors contain over a million instances, significantly slowing down the explanation generation process. Instead, we use the K-means implementation from the SHAP library to summarize the training data, as suggested in the documentation. MHCXAI accepts peptides and MHC alleles as input but generates test samples only for peptides from the SHAP package, as we focus solely on peptide explanations in this study. Each of these sample peptides is passed to the MHC class I predictor along with the allele to generate predictions. For binding affinity (BA) prediction, the BA values were converted to probabilities using the formula ${p}_{BA}=1-{\log }_{50000} \, BA$. The sample peptides and their corresponding predictions are then passed to the SHAP module to generate explanations for peptides.

LIME^10^ is an XAI technique that can be applied to complex machine learning models, including neural networks (NN). It locally replaces the intricate model with a simpler one, such as a linear regression model. LIME generates numerous perturbations of the original peptide sequence by mutating random positions, weighting these perturbations based on their ‘closeness’ to the original peptide to ensure that drastic perturbations have little impact. It then employs the simpler model to learn the mapping between the perturbations and any change in the output label. This process enables LIME to identify which positions are most crucial to the classification decision. The attribution values generated by LIME can be positive or negative, and we utilize the LIME package (https://github.com/marcotcr/lime) for explanation generation. For LIME, the number of samples to generate was set to 25,000 to minimize variance in LIME values. Similar to SHAP, for LIME, MHCXAI generates samples only for peptides using LIME package modules. These samples, along with alleles, are passed to the MHC class I predictor to generate predictions. For binding affinity (BA) prediction, the BA values were converted to probabilities using the formula ${p}_{BA}=1-{\log }_{50000} \, BA$. The sample peptides and their corresponding predictions are then passed to the LIME module to generate explanations.

For both XAIs, we found that providing all training peptides creates more accurate explanations than providing allele specific binding peptides (see Supplementary Note 7 and Supplementary Fig. S8).

### Validity of the explanations

To validate the explanations provided by SHAP and LIME, a comparison with ground truth is necessary. In this context, ground truth refers to the peptide residues that contribute the most to binding with the MHC molecule. Experimentally, this determination is made through Alanine-scanning mutagenesis^61^, where each residue in the ligand peptide is individually mutated to alanine. However, given the resource-intensive nature of the experimental approach, determining the crucial peptide positions can be independently achieved using BAlaS model^45,46^.

We obtained 250 PDB structures of peptide-MHC bound complexes, covering 40 MHC (HLA) alleles with peptides of length 9. Using BAlaS, we generated ΔΔ*G* for peptide positions for all 250 PDB structures. LIME and SHAP explanations were generated for all the investigated predictors for these 250 peptide-MHC allele pairs. For each peptide-MHC pair, we compared the LIME/SHAP explanation to corresponding vector of ΔΔ*G*.

### Explainable AI metrics

In order to evaluate quality of explanations, various metrics have been proposed^58,66,67^. Here, we consider two XAI metrics - Consistency and Stability.

Consistency: This metric captures whether explanations stay the same across similarly performing predictors^58,66^. Ideally, if models produce similar outputs, they should be focusing on similar features of the data when making predictions. Therefore, for a model agnostic XAI like LIME or SHAP, we expect replacing the black-box predictor with another predictor with comparable performance would produce similar explanations for any given input.

Stability: This metric assesses the extent of similarity between explanations for similar peptide instances^58^. To identify similar peptide instances, we cluster the input peptides for HLA-A*02:01 using GibbsCluster-2.0^62^, and we expect peptides belonging to the same cluster are similar. GibbsCluster-2.0 is a tool that aligns and clusters peptides in an unsupervised manner such that it maximizes the average Kullback–Leibler Distance (KLD) across the clusters. It accepts *λ* parameter ranging from 0 to 1 which represent penalty for intercluster similarity. Since we are interested in clustering binding peptides of an allele, we expect them to be largely homogeneous and want to detect subtle difference in patterns. To cluster such data, it is recommended to set *λ* to a very low value^62^ (*λ* = 0.05). However, this choice leads to overlapping clusters, where the Kullback–Leibler Distance (KLD) of a peptide within its cluster and across clusters is similar. To test stability, we select the most unrelated cluster pairs for further analysis. GibbsCluster-2.0 provides a position-specific scoring matrix (PSSM) for each cluster, generated using peptides within that cluster. We compare the PSSM of each cluster with others to identify unrelated cluster pairs. In the peptide clusters, we sampled 100 binding peptides and generated explanations for them. To assess the similarity of the explanations, we calculate the Euclidean distance between the explanations for intracluster and intercluster peptides. We limit the number of sampled peptides to 100 per cluster, as sampling more to generate explanations and calculate distances for all of them would be computationally expensive. We expect that the Euclidean distance for intracluster peptide explanations should be lower than the Euclidean distance for intercluster peptide explanations.

### Reporting summary

Further information on research design is available in the Nature Portfolio Reporting Summary linked to this article.

### Supplementary information

Supplementary Information

Description of Additional Supplementary Files

Supplementary Data 1-12

Reporting Summary

## Data Availability

The MHC-Bench dataset, MHC-Bench-v2 dataset and explanations reported in the study are available at https://github.com/PRBorole/MHCXAI. The other data required to recreate figures in the manuscript has been provided in Supplementary material. The numerical data behind the figures can be found in Supplementary Data 1–12.

## Code availability

Code for the explanation framework is available at the following url: https://github.com/PRBorole/MHCXAI.
