# Supplementary material for: Building trust in deep learning-based immune response predictors with interpretable explanations
Source: Commun Biol. 2024 Mar 6;7:279. doi: 10.1038/s42003-024-05968-2 (PMC10917751; doi:10.1038/s42003-024-05968-2)
Supplement: Supplementary file 1 — Supplementary Information [file 42003_2024_5968_MOESM1_ESM.pdf]

Supplementary Text

Building Trust in Deep Learning-based Immune Response Predictors with Interpretable Explanations

Piyush Borole, Ajitha Rajan

Supplementary Note 1: Introduction

In this document, we look at additional experiments that support the results in the main manuscript. Firstly, we look at additional benchmarking of the predictors by looking at a variant of the benchmark dataset and evaluate them on variety of metrics.

Furthermore, we show that explanations for alleles can also be generated using LIME [1] and SHAP [2] and global explanations can be formed by aggregation of attribution values. We also demonstrate that LIME and SHAP explanations are mostly correlated, and SHAP feature attribution values shows correlation while LIME attribution values are independent.

While BAlaS provides an important alternative to resource intensive Alanine-scanning Mutagenesis [3] to identify peptide residues contributing to binding to MHC molecule, it suffers two major limitations. The first limitation is that the energy calculations BAlaS does over the PDB structure of bound peptide-MHC allele molecule is seemingly affected by the resolution of the PDB structure. Here we demonstrate examples where difference in resolution marks a residue to be contributing to binding in one resolution while not contributing to binding in another resolution. The second major limitation is that BAlaS calculates energies by replacing the residue in peptide with alanine. However, if the input peptide contains alanine residue, the contribution of this residue cannot be calculated by BAlaS.

Next, we show the GibbsCluster [4] report of clustering input peptides for HLA-A*02:01 used to test stability of the explanations generated by LIME and SHAP. We also provide supplementary tables reporting the effect size and p-values. Supplementary Data 4 and 6 reports effect size in terms of Pearson’s r and Supplementary Tables 1-3 reports effect size using Cohen’s d.

The quality of explanations depends on the choice of certain parameters. LIME and SHAP are perturbation based XAI techniques, meaning that they generate test samples by mutating the original input peptide to evaluate the predictor and generate an explanation. The number of test samples to be generated (or the number of times the model should be evaluated) is one of the parameters that can be passed to the XAI techniques. If the model is not evaluated sufficiently, the attribution values display high variance. This variance reduces as the number of mutated samples increases. To find the right number of mutated samples for reducing variance, we set up an evaluation where we generate explanations for a peptide by varying the number of mutated samples the XAI technique should produce.

To generate these mutated samples, LIME and SHAP relies on the training data used to train predictor. Here, we explore if there is any difference in explanations generated if we provide training data covering all peptides across alleles or training peptides specific to the MHC allele of interest. We test the validity of the explanations generated by providing these two training datasets.

We also look at the nature of these explanations and the congruence between the explanations generated by LIME and SHAP.

Supplementary Note 2: Additional benchmarking of predictor performance

In the main manuscript, we assessed performance of the predictors using the MHC-Bench dataset. However, as mentioned earlier, MHC-Bench contains Peptide-MHC pairs that were not seen during training, though the peptides themselves may have been part of the training data for the predictors. Here, we present additional analysis by excluding training peptides from the MHC-Bench dataset, creating a new dataset referred to as "MHC-Bench-v2".

After removing training peptides, we observed that, for 36 MHC alleles, there were no binder peptides available. We excluded these MHC alleles from our analysis, as the calculation of AUROC and AUPRC scores is not possible with only the negative or non-binder class. The MHC-Bench-v2 dataset comprises of 79 alleles, 1,282,057 unique peptides, and 1,666,269 peptide-MHC combinations. We calculated AUROC and AUPRC scores per allele which are provided in Supplementary Data 7 and 8. The percentage of binding peptides per allele is reported in Supplementary Data 9. In Supplemental Figure S1a, the AUROC v1 scores represent the scores on the original MHC-Bench dataset, and AUROC v2 scores represent the scores on the MHC-Bench-v2. The performances appear to be comparable to the performance on the MHC-Bench. In Supplemental Figure S1b, the AUPRC v1 scores represent the scores on the original MHC-Bench dataset, and AUPRC v2 scores represent the scores on the MHC-Bench-v2. With AUPRC, there is a noticeable drop in performance. Nevertheless, the performance of the predictors is still comparable and not significantly different (Kruskal-Wallis p-value: 0.45, H-statistic: 4.67).

Additionally, considering that the threshold for HLA alleles varies [7], many predictors recommend using a percentile score for the classification of peptides into binders or non-binders. However, not all predictors provide the percentile score. Among our selected predictors, MHCfovea and NetMHCpan offer %Rank, whereas MHCflurry provides the percentile only for the final presentation score (referred to as MHCflurry-PS in our manuscript). TransPHLA does not provide %Rank (or percentile) for the peptides but a binding probability. This limitation hinders our comparison across all predictors. Nonetheless, we compare the performance of the four predictors based on %Rank (or percentile). We set 0.5% as the cut-off for binders, as recommended in the original publications of the predictors, and calculate the F1 score per allele (results in Supplementary Data 10). Supplemental Figure S1c shows the distribution of the F1 score for the four predictors. We find that the performance of the predictors is comparable and not significantly different (Kruskal-Wallis p-value: 0.19, H-statistic: 4.73).

These two analyses indicate that the performances of the predictors are comparable across metrics and scoring methods.

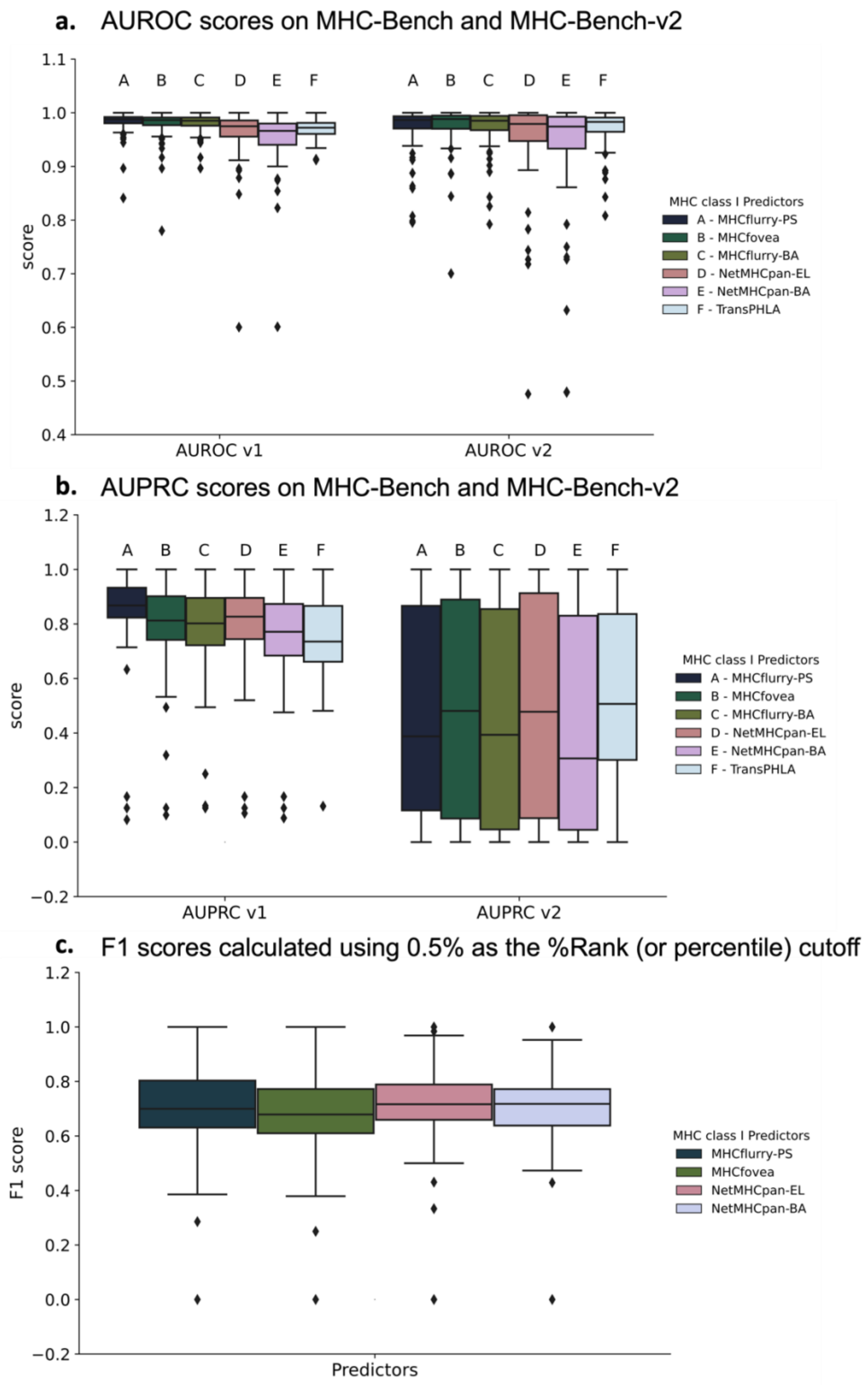

Supplemental Figure S1: Additional benchmarking results. **a)** AUROC scores for the predictors on MHC-Bench and MHC-Bench-v2. **b)** AUPRC scores for the predictors on MHC-Bench and MHC-Bench-v2. **c)** F1 scores for the predictors on MHC-Bench calculated using %Rank (or percentile in MHCflurry-PS).

Supplementary Note 3: Explanations for MHC Allele (TransPHLA)

Similarly, to peptide explanations, we can also generate explanations for alleles in MHC class I predictors that take MHC molecules as input sequences. For example, TransPHLA accepts both peptide and MHC molecule pseudo-sequences as input. A pseudo-sequence of an MHC molecule consists of 34 residues forming the binding pocket of the MHC molecule's alpha subunit. To generate explanations for MHC alleles, we use the same framework but modify it such that the input peptide remains constant, while the MHC allele pseudo-sequence is perturbed. In Supplemental Figure S2, we demonstrate that both LIME and SHAP can generate explanations for the HLA-A*02:01 allele sequence for TransPHLA.

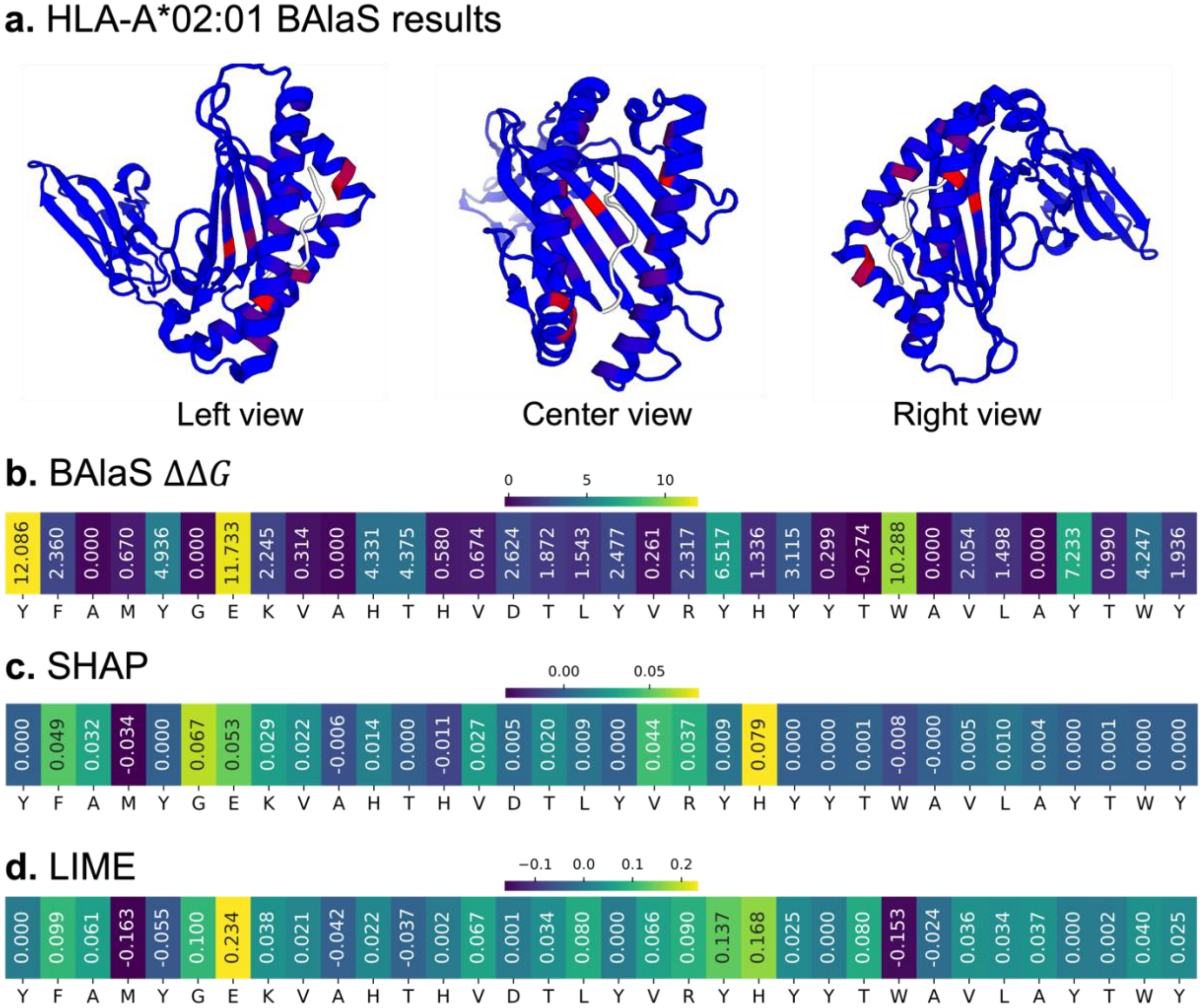

Supplemental Figure S2. Explanation for MHC allele HLA-A*02:01 for TransPHLA. **a)** BAlaS result indicating allele residues contributing to binding (marked in red) from left, center, and right view of the PDB structure. The molecule in white is the ligand peptide ITDQVPFSV. **b-d)** ΔΔG, calculated using BAlaS [3,5], for 34 pseudo-sequence positions of HLA-A*02:01 **(b)** along with SHAP **(c)** and LIME **(d)** explanations for those positions. Explanations for TransPHLA can be generated as it accepts allele input as a sequence of amino acids.

Supplementary Note 4: Global Explanations as Aggregation of Attribution Values

Local instance-based explanations do not eliminate the need for global explanations; each has its role. For example, global explanations help in understanding commonly presented peptide patterns for a tumor, which can be utilized in the development of a cancer vaccine. It is essential to note that local instance-based explanations can be aggregated to generate a global explanation. Consider Supplemental Figure S3a, c, which displays the distribution of SHAP and LIME attribution values for all amino acids at peptide positions P1, P2, P8, and P9 for HLA-A*02:01 for MHCflurry-PS. This distribution serves as a global explanation across all peptides for the MHC allele HLA-A*02:01. The heatmaps in Supplemental Figure S3b, d are produced by averaging all the values from plots like Supplemental Figure S3a, c for all positions. We observe that the SHAP attribution values for an amino acid have a wider range of values, whereas LIME attribution values for the corresponding amino acid tend to have a very narrow range of values. This difference could explain why LIME produces more stable and consistent explanations than SHAP.

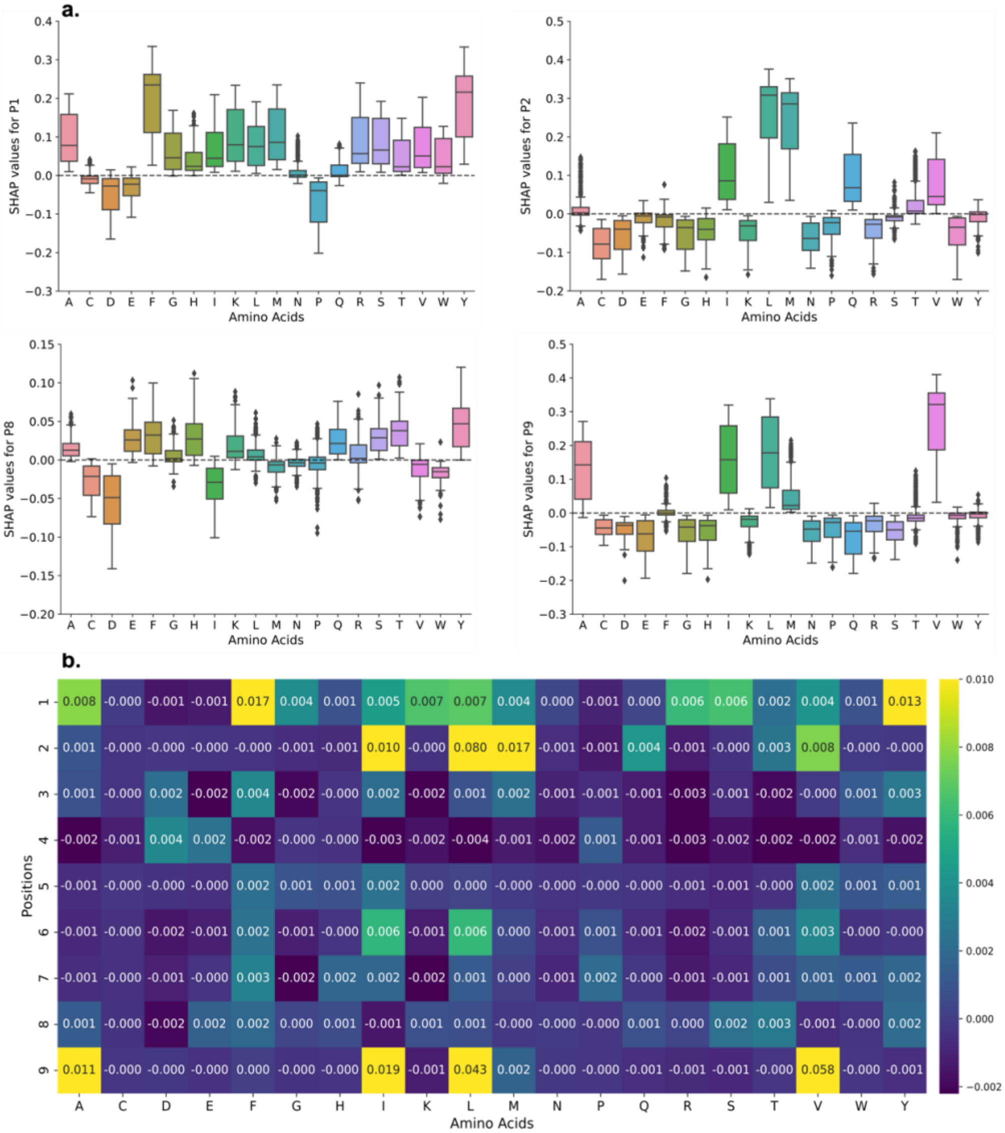

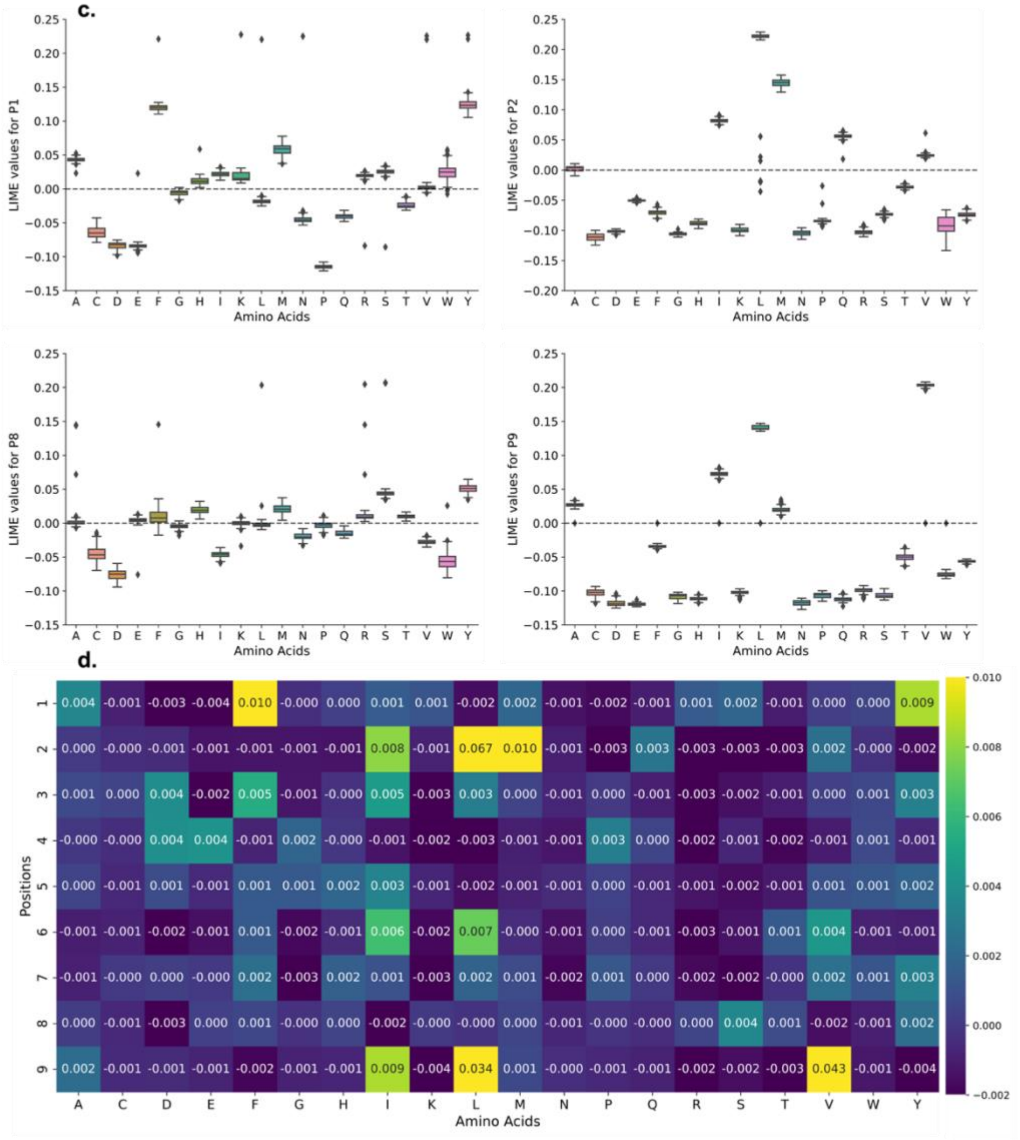

Supplemental Figure S3. Aggregation of SHAP and LIME attribution values to form global explanation. **a)** SHAP attribution values distribution for MHC allele HLA-A*02:01 for all amino acids at N- and C- terminus (P1, P2, P8 and P9) for peptide. **b)** Heatmap of average SHAP values for all amino acids at all peptide positions (P1-P9) for HLA-A*02:01. **c)** LIME attribution values distribution for MHC allele HLA-A*02:01 for all amino acids at N- and C- terminus (P1, P2, P8 and P9) for peptide. **d)** Heatmap of average LIME values for all amino acids at all peptide positions (P1-P9) for HLA-A*02:01. SHAP attribution values for an amino acid tends to have wide distribution compared to corresponding LIME attribution values.

Supplementary Note 5: Limitation of BAlaS

BAlaS [3,5] offers a cost-effective alternative to the expensive Alanine-scanning mutagenesis [3] for calculating the free energy of interaction (ΔΔG) from the PDB structure of a peptide ligand bound to an MHC molecule. This ΔΔG can help identify peptide residues contributing to binding. However, BAlaS has two main limitations: ΔΔG calculations are influenced by the resolution of the PDB structure, and the contribution of alanine residues cannot be determined. Supplemental Figure S4 illustrates the first limitation, where for the same peptide-MHC allele pair, one PDB structure highlight certain residues as 'hot' residues, while another structure may not. In Supplemental Figure S4b, it is evident that in structure 5D2L, residue N has a negative ΔΔG, whereas for the same peptide-MHC allele pair in structure 3GSO, residue N has a neutral effect. The second limitation is demonstrated in Supplemental Figure S5, where the residue at peptide position P2 in both structures is alanine. Although P2 is an anchor residue appropriately highlighted by the SHAP explanation, BAlaS is unable to quantify the contribution of this position.

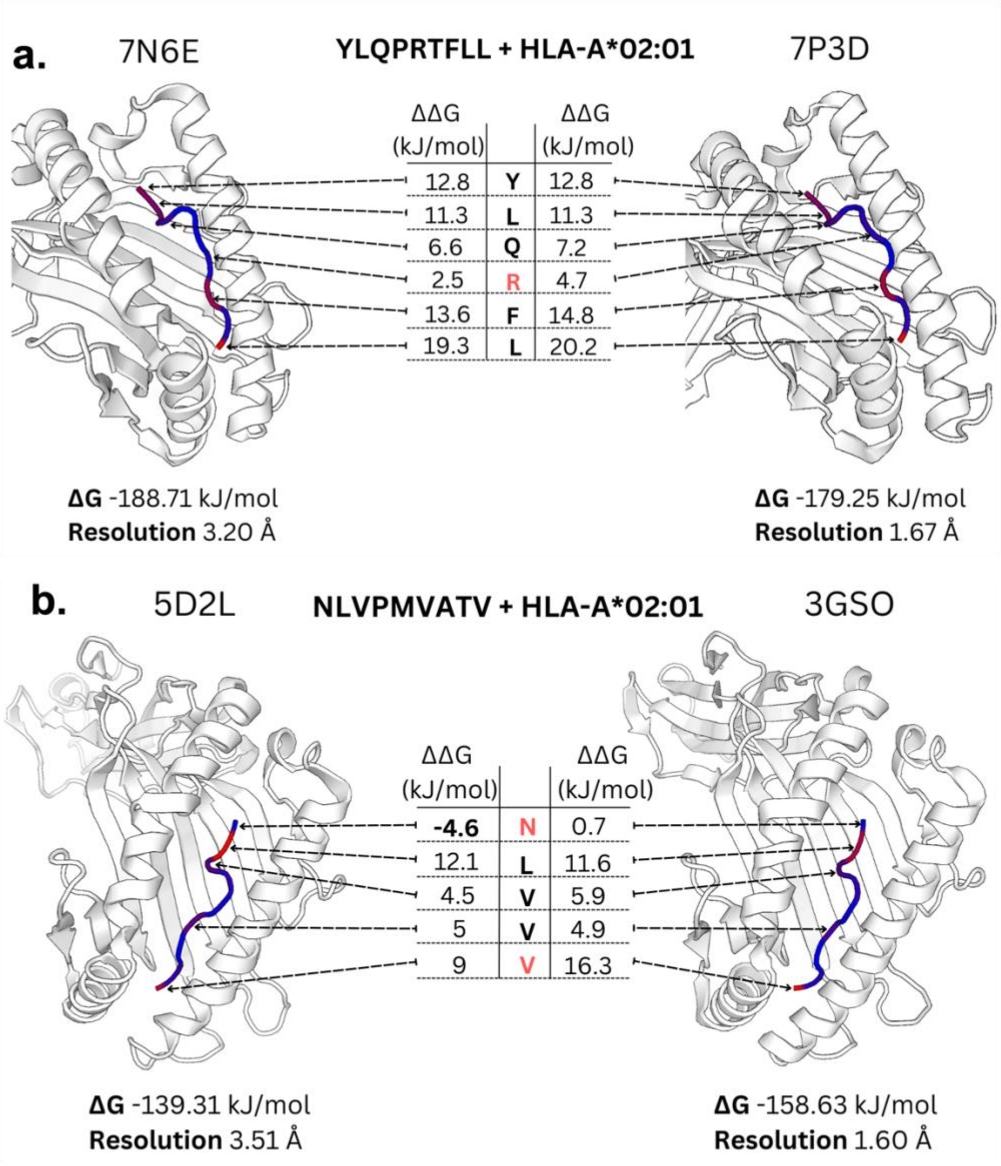

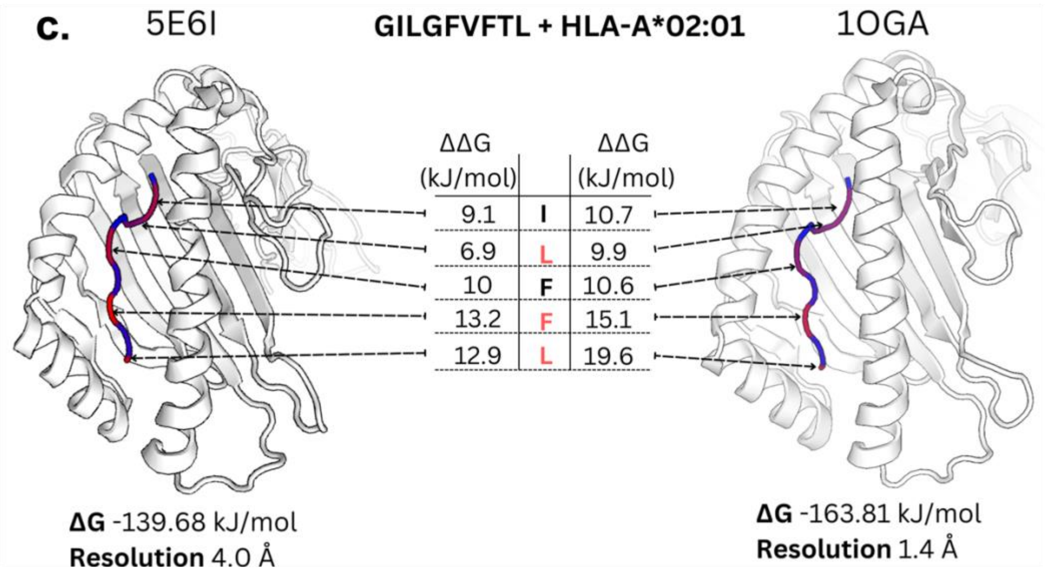

Supplemental Figure S4. Impact of resolution of PDB structure on $\Delta\Delta G$ calculation. In all **a, b** and **c,** there are two PDB structure of different resolutions showing peptide bound to MHC allele. The residues that have relatively large difference in $\Delta\Delta G$ are highlighted in red. The difference in free energy (ΔG) due to difference in resolution can be more than 20 kJ/mol. In **a**, residue R is not highlighted as 'hot' residue in coarser resolution PDB structure whereas for 7P3D, $\Delta\Delta G > 4.18\ kJ/mol$ indicating that it is contributing to binding. In **b,** we see that the residue N have negative $\Delta\Delta G$ for PDB structure 5D2L whereas for the finer PDB structure 3GSO, the same residue has no impact. The peptide-HLA models are created by BAlaS online.

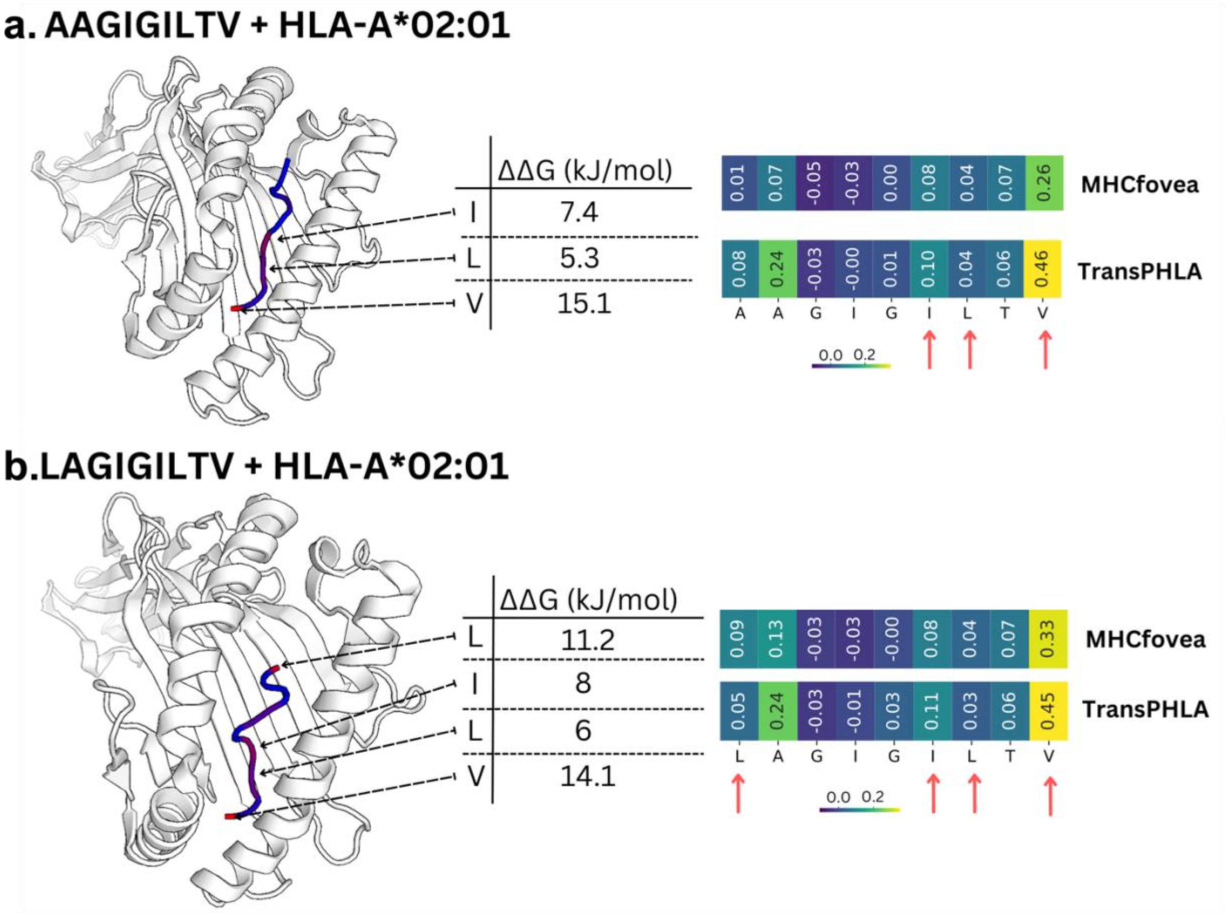

Supplemental Figure S5. Additional examples of comparison between BAlaS ΔΔG and SHAP explanations and demonstration of limitation of BAlaS. Here, peptides AAGIGILTV **(a)** and LAGIGILTV **(b)** are both binding peptides to MHC allele HLA-A*02:01 with only first amino acid difference. **a)** The BAlaS identifies that for peptide AAGIGILTV, peptide positions P6, P7 and P9 are important for the binding. The two heatmaps are SHAP explanations for MHCfovea and TransPHLA which correctly classified the peptide as binder. The explanations reveal that while P9 is important position, they do not place high importance to other important positions P6 and P7. **b)** For peptide LAGIGILTV, P1, P6, P7 and P9 are important residues contributing to the binding. The two heatmaps are SHAP explanations for MHCfovea and TransPHLA which correctly classified the peptide as binder. The explanations reveal that while P9 is important residue, P6 and P7 are not considered highly important for classification even though they contribute strongly to binding. We see that in **(b)** 'L' has very high (ΔΔG) indicating the importance of the position. However, neither of the models highlights this to be an important position. TransPHLA indicates P2 as important position contributing to binding which is not indicated by BAlaS. This is important as P2 is one of the primary anchor positions. However, as BAlaS replaces amino acids with 'A' to calculate (ΔΔG), we cannot calculate the contribution of 'A' towards binding (For example at position P2 in LAGIGILTV). The peptide-HLA models are created by BAlaS online

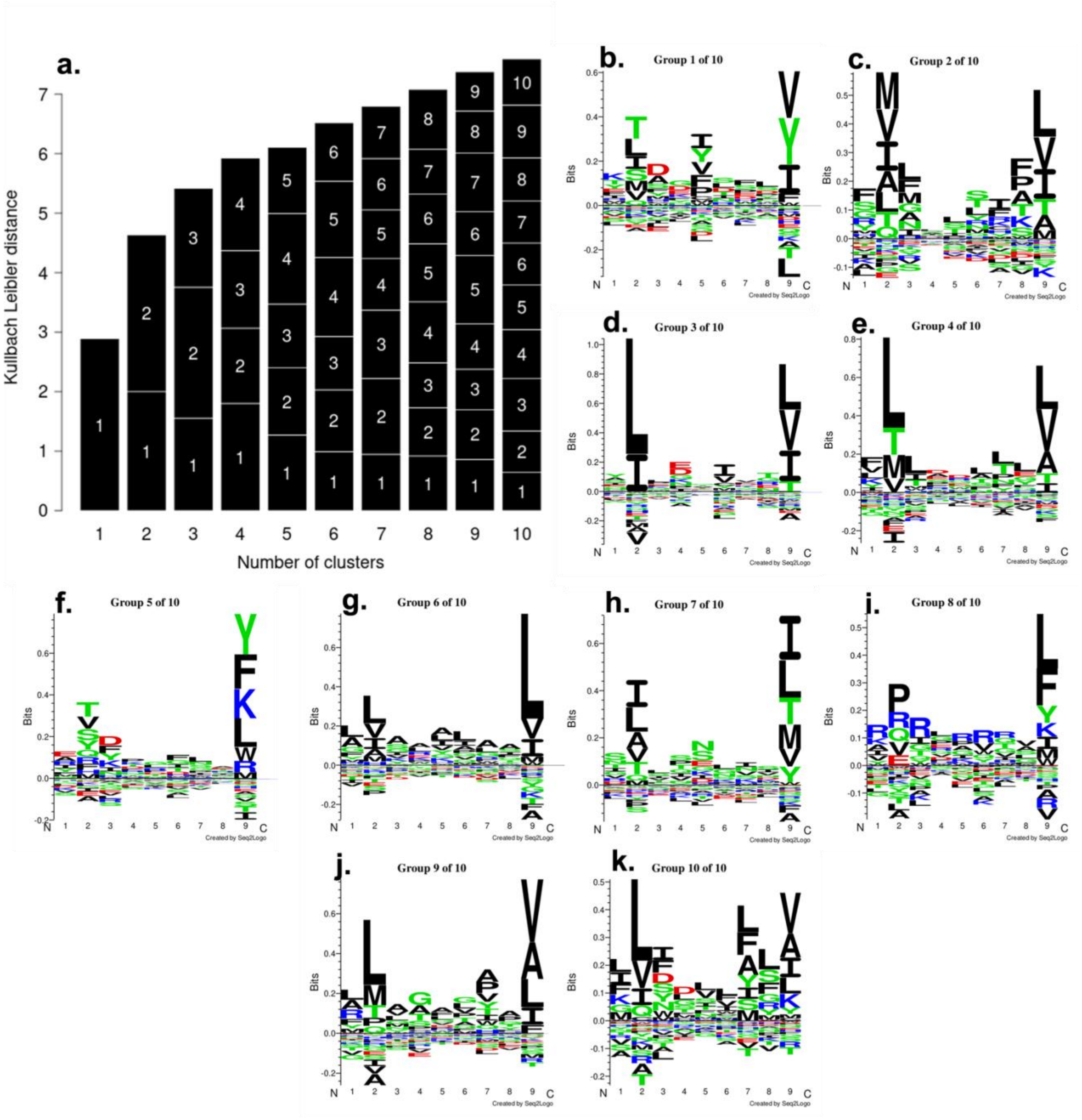

Supplemental Figure S6. GibbsCluster [4] report for MHC allele HLA-A*02:01 peptides. **a)** Kullbach-Leibler Distance (KLD) when peptides are clustered into 1-10 clusters. The KLD increases with the number of clusters. The number of clusters with maximum average KLD (Here 10 clusters) is recommended as best clustering result [4]. The length of labelled section in each column represents the size of that cluster. For 10 clusters, all the clusters contain nearly the same number of peptides as seen by similar length of sections. **b-k)** The motifs of the peptides present in 10 clusters.

Supplementary Note 6: Convergence of Explanations

To generate explanations, LIME produces perturbed samples where it replaces an amino acid with another amino acid sampled from a distribution derived from the training peptide dataset. Similarly, SHAP evaluates the model by replacing the amino acid in the test peptide with an amino acid from the training peptide dataset. As the number of test samples generated by LIME and SHAP increases, the variance of the attribution value decreases. To test this, we generated explanations for 20 peptides (10 binders, 10 non-binders) for HLA-A*02:01 using LIME and SHAP. We considered explanations generated with 25,000 perturbed samples as the baseline. Next, we generated explanations for all 20 peptides using 1,000 – 25,000 perturbed samples, and the Euclidean distance is calculated from the baseline. In Supplemental Figure S7, we see that the Euclidean distance approaches zero for SHAP (Supplemental Figure S7a) rapidly as the number of perturbed samples increases until 10,000 samples. While for LIME explanations, Euclidean distance initially drops rapidly until 7,500 perturbed samples, after which the value plateaus and does not approach zero (Supplemental Figure S7b). In Supplemental Figure S7, each dot represents the average Euclidean distance calculated for 20 peptide explanations. The errorbar indicates the variance in Euclidean distance, and as the number of perturbed samples increases, the errorbar reduces, indicating a reduction in variance in both LIME and SHAP attribution values.

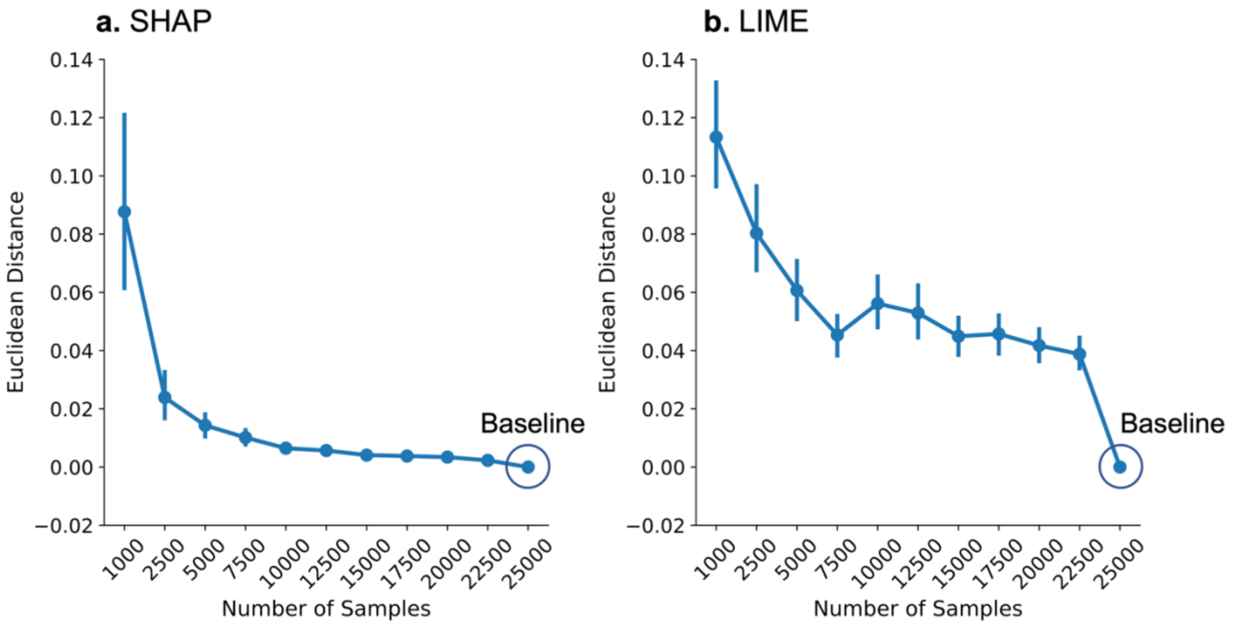

Supplemental Figure S7. Convergence of explanations. LIME and SHAP generate explanations by evaluating the model over perturbed samples. The attribution values start to converge from 10,000 samples for SHAP **(a)** and 7500 samples for LIME **(b)**. The SHAP attribution values comes very close to baseline (attribution values generated using 25,000 samples) but LIME attribution values do not eventually converge to baseline. However, as the number of samples increase, the error bar size reduces in both SHAP and LIME.

Supplementary Note 7: Impact of Training Data

Since generating explanations requires training data, we also explored whether the explanation changes if we provide the entire training data across all alleles or just training data specific to the allele of interest. From our PDB dataset of bound peptide-MHC structures, we selected structures for alleles HLA-A*02:01, HLA-B*07:02, and HLA-B*35:01. These alleles have many peptides-MHC bound PDB structures (105, 9, and 11 structures for HLA-A*02:01, HLA-B*07:02, and HLA-B*35:01, respectively). We used the ΔΔG for peptide positions obtained from BAlaS for these structures and generated LIME and SHAP explanations using the ‘All training peptides’ dataset and ‘Allele-specific peptides’ dataset. For each structure, the Pearson correlation coefficient between ΔΔG and explanations is calculated and plotted in Supplemental Figure S8a, b for SHAP and LIME, respectively. We found that SHAP explanations generated using ‘Allele-specific peptides’ are similar to explanations generated using ‘All training peptides’ and are no more or less correlated to the BAlaS ΔΔG. On the contrary, we observed that limiting training data to allele-specific peptides could be detrimental to the accuracy of the LIME explanations than the explanations generated using ‘All training peptides’.

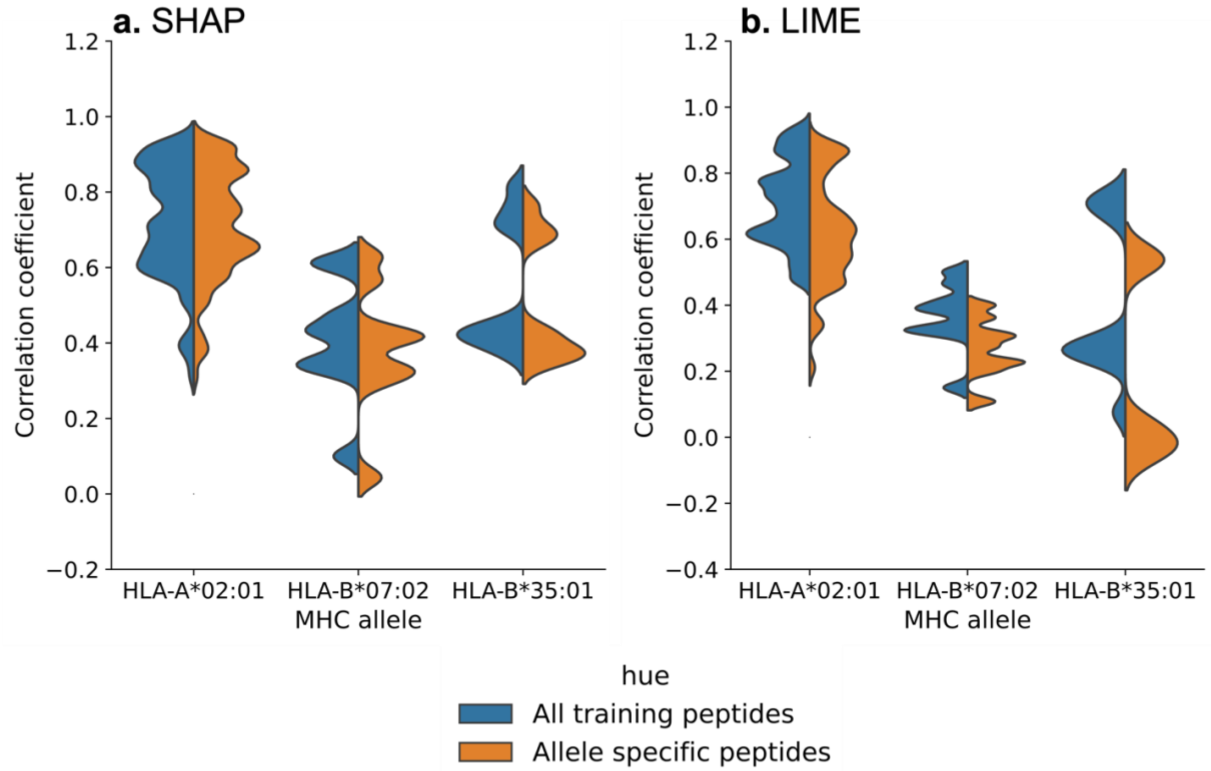

Supplemental Figure S8. Impact of limiting training data on SHAP and LIME explanations. Validity is tested for two sets of explanations generated using ‘All training peptides’ and ‘Allele specific peptides’ against ΔΔG values. **a)** SHAP explanations are not affected either positively or negatively by limiting training data to alleles specific peptides. This is indicated by similar distribution of correlation coefficient between ΔΔG and attribution values generated from two training datasets. **b)** On the contrary, LIME explanations become unreliable when training data is limited to allele specific peptides as seen by difference in correlation coefficient distribution between the two training datasets. The correlation coefficients are reported in Supplementary Data 11.

Supplementary Note 8: Properties of Explanations

LIME and SHAP produce stable and consistent explanations for MHC class I predictors which mostly agree with the independently derived important positions using BAlaS. We wanted to further explore qualities and features of the explanations. First, we explored if the SHAP and LIME explanation for an MHC class I predictor (MHCflurry-PS) agree with each other. Supplemental Figure S9a shows distribution of correlation coefficient between LIME and SHAP for 9 MHC class I allele. We find that the LIME and SHAP explanations are highly correlated. We also explored if the attribution values of explanations are dependent on each other. Supplemental Figure S9b are correlation heatmaps of SHAP/LIME attribution values of peptide positions. For all 3 alleles, SHAP attribution values interdependent (especially for anchor positions P1, P2 and P9) while LIME attribution values were independent. This is possible when SHAP produces test samples from a distribution that has dependent features [9] whereas LIME samples independently for each peptide position from the amino acid frequency generated from training data.

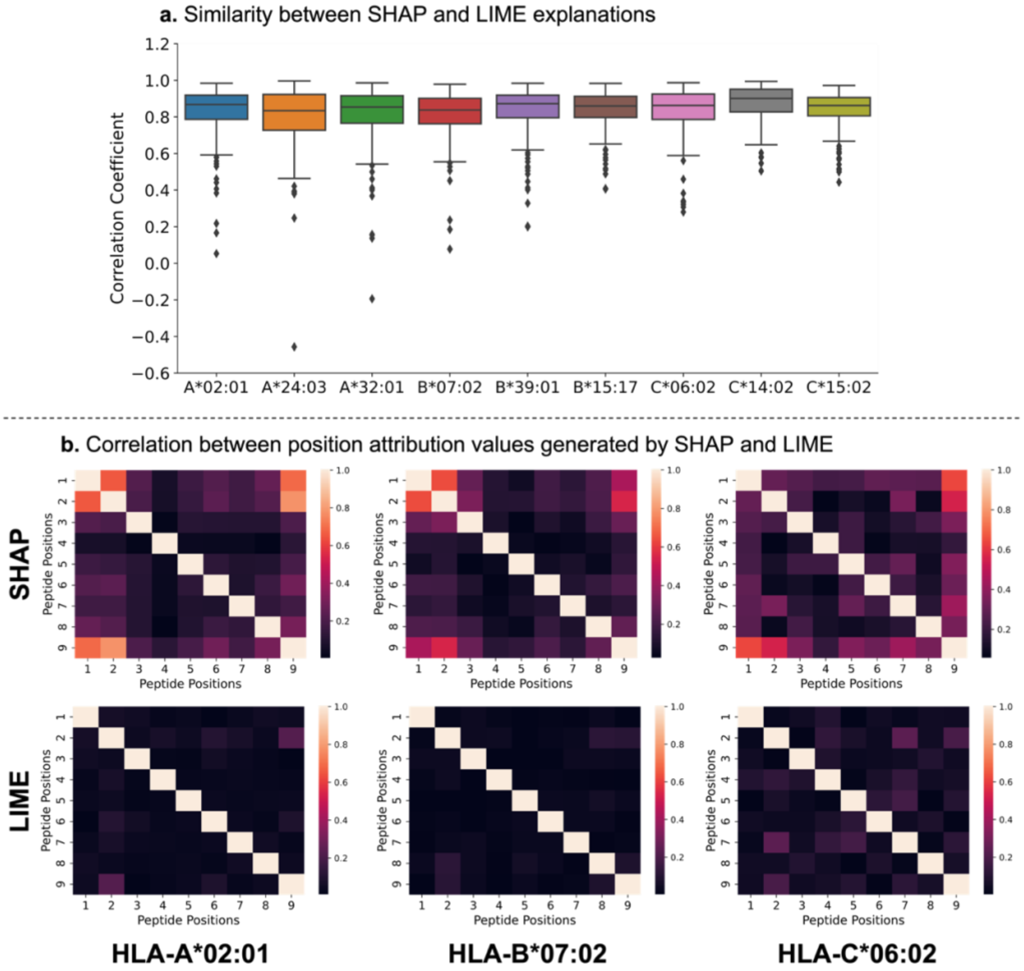

Supplemental Figure S9. Comparison of explanations produced by SHAP and LIME for same input samples and correlation between the attribution values for peptide positions. **a)** The LIME and SHAP explanations are highly correlated for the nine MHC class I alleles. **b)** Correlation heatmaps for attribution values among all peptide positions indicates that SHAP attribution values can be correlated whereas LIME attribution values tend to be independent. The correlation coefficient values are reported in Supplementary Data 12.

Supplementary Tables

Cohen's d is calculated as follows:

$$d = \frac{\overline{x_1} - \overline{x_2}}{s}$$

$$s = \sqrt{\frac{(n_1 - 1)s_1^2 + (n_2 - 1)s_2^2}{n_1 + n_2 - 2}}$$

Where, $\overline{x_1}, \& \overline{x_2}$ are mean of the two group, s is pool standard deviation, s_1^2 and s_2^2 are variances of two groups and n_1 and n_2 are number of samples in the groups.

Supplementary Table 1: Kruskal Wallis test results: p-values, H-statistic and effect size Cohen's d for SHAP explanations generated for testing Consistency.

HLA	p-value	H-statistic	Cohen's d
HLA-A*02:01	1.067e-24	105.267	1.369
HLA-A*24:03	2.709e-18	76.090	0.944
HLA-A*32:01	8.583e-34	146.822	1.613
HLA-B*07:02	2.235e-33	144.920	1.443
HLA-B*39:01	6.167e-10	38.267	0.741
HLA-B*15:17	9.774e-26	110.004	1.177
HLA-C*06:02	4.157e-40	175.725	1.717
HLA-C*14:02	2.302e-56	250.240	2.196
HLA-C*15:02	1.143e-39	173.713	1.888

Supplementary Table 2: Kruskal Wallis test results: p-values, H-statistic and effect size Cohen's d for LIME explanations generated for testing Consistency.

HLA	p-value	H-statistic	Cohen's d
HLA-A*02:01	4.856e-65	290.046	2.754
HLA-A*24:03	3.792e-26	111.881	1.174
HLA-A*32:01	1.091e-48	215.040	1.770
HLA-B*07:02	1.027e-57	256.435	1.364
HLA-B*39:01	2.366e-53	236.425	1.565
HLA-B*15:17	4.785e-57	253.369	2.019
HLA-C*06:02	4.284e-57	253.589	2.273
HLA-C*14:02	3.016e-62	277.228	2.663
HLA-C*15:02	1.057e-61	274.728	2.792

Supplementary Table 3: Kruskal Wallis test results: p-values, H-statistic and effect size Cohen's d for SHAP and LIME explanations generated for testing Stability.

Pairs	Order	SHAP			LIME		
		p-val.	H	Cohen's d	p-val.	H	Cohen's d
c2, c5	Intracluster$_L$ - Intercluster	1.552e-18	365.819	0.842	9.022e-29	123.863	0.487
	Intracluster$_R$ - Intercluster	0.964	0.001	0.022	1.231e-09	36.918	0.283
c3, c5	Intracluster$_L$ - Intercluster	1.245e-145	660.362	1.014	1.302e-125	568.320	0.854
	Intracluster$_R$ - Intercluster	5.611e-07	25.041	0.232	4.379e-77	345.342	0.869
c5, c6	Intracluster$_L$ - Intercluster	0.119	2.425	0.077	1.104e-38	169.202	0.463
	Intracluster$_R$ - Intercluster	3.758e-199	906.517	0.757	1.129e-87	393.974	0.470
c5, c9	Intracluster$_L$ - Intercluster	2.728e-09	35.368	0.241	1.878e-87	392.958	0.723
	Intracluster$_R$ - Intercluster	0.0	1470.400	0.847	4.420e-167	759.004	0.632
c5, c10	Intracluster$_L$ - Intercluster	0.064	3.415	0.099	3.654e-56	249.319	0.571
	Intracluster$_R$ - Intercluster	1.431e-133	604.910	0.581	5.690e-24	101.951	0.259
c3, c8	Intracluster$_L$ - Intercluster	1.424e-80	361.358	1.077	7.986e-57	252.348	0.819
	Intracluster$_R$ - Intercluster	1.152e-05	19.239	0.292	6.720e-21	87.94	0.654

Supplementary References

1. Ribeiro, M.T., Singh, S., Guestrin, C.: "why should I trust you?": Explaining the predictions of any classifier. In: Proceedings of the *22nd ACM SIGKDD International Conference on Knowledge Discovery and Data Mining, San Francisco, CA, USA*, August 13-17, 2016, pp. 1135–1144 (2016)
2. Lundberg, S.M., Lee, S.-I.: A unified approach to interpreting model predictions. *Advances in neural information processing systems* **30** (2017)
3. Ibarra, A.A., Bartlett, G.J., Hegedus, Z., Dutt, S., Hobor, F., Horner, K.A., Hetherington, K., Spence, K., Nelson, A., Edwards, T.A., et al.: Predicting and experimentally validating hot-spot residues at protein–protein interfaces. *ACS chemical biology* **14**(10), 2252–2263 (2019)
4. Andreatta, M., Alvarez, B., Nielsen, M.: Gibbscluster: unsupervised clustering and alignment of peptide sequences. *Nucleic acids research* **45**(W1), 458–463 (2017)
5. Wood, C. W., Ibarra, A. A., Bartlett, G. J., Wilson, A. J., Woolfson, D. N., & Sessions, R. B. (2020). BAlaS: fast, interactive and accessible computational alanine-scanning using BudeAlaScan. *Bioinformatics*, ***36***(9), 2917-2919.
6. Cunningham, B. C., & Wells, J. A. (1989). High-resolution epitope mapping of hGH-receptor interactions by alanine-scanning mutagenesis. *Science*, ***244***(4908), 1081-1085.
7. Reardon, B., Koşaloğlu-Yalçın, Z., Paul, S., Peters, B. and Sette, A., 2021. Allele-specific thresholds of eluted ligands for T-cell epitope prediction. *Molecular & Cellular Proteomics*, *20*.
8. Aas, K., Jullum, M., & Løland, A. (2021). Explaining individual predictions when features are dependent: More accurate approximations to Shapley values. *Artificial Intelligence*, ***298***, 103502.
