# Supplementary material for: Building trust in deep learning-based immune response predictors with interpretable explanations
Source: Commun Biol. 2024 Mar 6;7:279. doi: 10.1038/s42003-024-05968-2 (PMC10917751; doi:10.1038/s42003-024-05968-2)
Supplement: Supplementary file 2 — Description of Additional Supplementary Files [file 42003_2024_5968_MOESM2_ESM.pdf]

Description of Additional Supplementary Files

File name: Supplementary Data 1
Description: AUROC scores per allele for all investigated MHC class I predictors on MHC-Bench.

File name: Supplementary Data 2
Description: AUPRC scores per allele for all the investigate MHC class I predictors on MHC-Bench.

File name: Supplementary Data 3
Description: Percent binding peptide-MHC pairs per allele for MHC-Bench.

File name: Supplementary Data 4
Description: Pearson r (correlation coefficient) between SHAP/ LIME explanations and ΔΔG. Values plotted in Figure 5b of the main article.

File name: Supplementary Data 5
Description: PDB structures list used to generate ground truth using BalaS.

File name: Supplementary Data 6
Description: Pearson r (Correlation coefficient) for SHAP /LIME explanations for testing consistency. Values plotted in Figure 6c of the main article.

File name: Supplementary Data 7
Description: AUROC scores per allele for all investigated MHC class I predictors on MHC-Bench-v2.

File name: Supplementary Data 8
Description: AUPRC scores per allele for all the investigate MHC class I predictors on MHC-Bench-v2.

File name: Supplementary Data 9
Description: Percent binding peptide-MHC pairs per allele for MHC-Bench-v2.

File name: Supplementary Data 10
Description: F1 scores per allele for all investigated MHC class I predictors.

File name: Supplementary Data 11
Description: Pearson r (Correlation Coefficient) between LIME/SHAP explanations generated using all training peptides and allele specific peptides and corresponding $\Delta\Delta G$. Values plotted in Supplemental Figure S8.

File name: Supplementary Data 12
Description: Pearson r (Correlation coefficient) between SHAP and LIME explanations. Values plotted in Supplemental Figure S9a.
