# Supplementary material for: Building trust in deep learning-based immune response predictors with interpretable explanations
Source: Commun Biol. 2024 Mar 6;7:279. doi: 10.1038/s42003-024-05968-2 (PMC10917751; doi:10.1038/s42003-024-05968-2)
Supplement: Supplementary file 4 — Reporting Summary [file 42003_2024_5968_MOESM4_ESM.pdf]

nature portfolio

Corresponding author(s): Piyush Borole, Ajitha Rajan

Last updated by author(s): 24/01/24

Reporting Summary

Nature Portfolio wishes to improve the reproducibility of the work that we publish. This form provides structure for consistency and transparency in reporting. For further information on Nature Portfolio policies, see our Editorial Policies and the Editorial Policy Checklist.

Statistics

For all statistical analyses, confirm that the following items are present in the figure legend, table legend, main text, or Methods section.

n/a	Confirmed	
☐	☒	The exact sample size (*n*) for each experimental group/condition, given as a discrete number and unit of measurement
☐	☒	A statement on whether measurements were taken from distinct samples or whether the same sample was measured repeatedly
☐	☒	The statistical test(s) used AND whether they are one- or two-sided<br>*Only common tests should be described solely by name; describe more complex techniques in the Methods section.*
☐	☒	A description of all covariates tested
☐	☒	A description of any assumptions or corrections, such as tests of normality and adjustment for multiple comparisons
☐	☒	A full description of the statistical parameters including central tendency (e.g. means) or other basic estimates (e.g. regression coefficient) AND variation (e.g. standard deviation) or associated estimates of uncertainty (e.g. confidence intervals)
☐	☒	For null hypothesis testing, the test statistic (e.g. *F*, *t*, *r*) with confidence intervals, effect sizes, degrees of freedom and *P* value noted<br>*Give P values as exact values whenever suitable.*
☒	☐	For Bayesian analysis, information on the choice of priors and Markov chain Monte Carlo settings
☒	☐	For hierarchical and complex designs, identification of the appropriate level for tests and full reporting of outcomes
☐	☒	Estimates of effect sizes (e.g. Cohen's *d*, Pearson's *r*), indicating how they were calculated

Our web collection on statistics for biologists contains articles on many of the points above.

Software and code

Policy information about availability of computer code

Data collection	No software was used for data collection.
Data analysis	MHC class I predictors - MHCflurry-2.0, NetMHCpan-4.1, MHCfovea, TransPHLA<br>Python-3.8<br>Python Packages - SHAP-0.41.0, Lime-0.2.0.1, SciPy-1.4.1, Seaborn-0.11.2, Matplotlib-3.4.3, Scikit-learn-1.2.1, Numpy-1.18.5<br>BAlaS (BudeAlaScan)-1.0, GibbsCluster-2.0

For manuscripts utilizing custom algorithms or software that are central to the research but not yet described in published literature, software must be made available to editors and reviewers. We strongly encourage code deposition in a community repository (e.g. GitHub). See the Nature Portfolio guidelines for submitting code & software for further information.

Data

Policy information about availability of data

All manuscripts must include a data availability statement. This statement should provide the following information, where applicable:

- Accession codes, unique identifiers, or web links for publicly available datasets
- A description of any restrictions on data availability
- For clinical datasets or third party data, please ensure that the statement adheres to our policy

We downloaded the training datasets for MHC class I predictors from their respective GitHub pages -
TransPHLA - https://github.com/a96123155/TransPHLA-AOMP/tree/master/Dataset
MHCflurry - https://data.mendeley.com/datasets/zx3kjzc3yx/3
MHCfovea - https://data.mendeley.com/datasets/c249p8gdzd/3
netMHCpan - https://services.healthtech.dtu.dk/suppl/immunology/NAR_NetMHCpan_NetMHCIIpan/

To create MHC-Bench dataset, we combined publicly available datasets from the MHC class I predictors from following links -
TransPHLA (External + Independent) - https://github.com/a96123155/TransPHLA-AOMP/tree/master/Dataset
MHCflurry (monoallelic benchmark) - https://data.mendeley.com/datasets/zx3kjzc3yx/3
netMHCpan - https://services.healthtech.dtu.dk/suppl/immunology/NAR_NetMHCpan_NetMHCIIpan/

PDB structures list was downloaded from MHC Atlas (http://mhcmotifatlas.org/home) Online webpage and the .pdb files were downloaded from PDB website (https://www.rcsb.org/)
The processing of these datasets is described in Methods section of the main article.

There is no restriction on data availability as all the data used for this study is open access available from the links provided. For this research no clinical datasets were used.

Human research participants

Policy information about studies involving human research participants and Sex and Gender in Research.

Reporting on sex and gender	*Use the terms sex (biological attribute) and gender (shaped by social and cultural circumstances) carefully in order to avoid confusing both terms. Indicate if findings apply to only one sex or gender; describe whether sex and gender were considered in study design whether sex and/or gender was determined based on self-reporting or assigned and methods used. Provide in the source data disaggregated sex and gender data where this information has been collected, and consent has been obtained for sharing of individual-level data; provide overall numbers in this Reporting Summary. Please state if this information has not been collected. Report sex- and gender-based analyses where performed, justify reasons for lack of sex- and gender-based analysis.*
Population characteristics	*Describe the covariate-relevant population characteristics of the human research participants (e.g. age, genotypic information, past and current diagnosis and treatment categories). If you filled out the behavioural & social sciences study design questions and have nothing to add here, write "See above."*
Recruitment	*Describe how participants were recruited. Outline any potential self-selection bias or other biases that may be present and how these are likely to impact results.*
Ethics oversight	*Identify the organization(s) that approved the study protocol.*

Note that full information on the approval of the study protocol must also be provided in the manuscript.

Field-specific reporting

Please select the one below that is the best fit for your research. If you are not sure, read the appropriate sections before making your selection.

☒ Life sciences ☐ Behavioural & social sciences ☐ Ecological, evolutionary & environmental sciences

For a reference copy of the document with all sections, see nature.com/documents/nr-reporting-summary-flat.pdf

Life sciences study design

All studies must disclose on these points even when the disclosure is negative.

Sample size	For testing validity, as only 250 PDB structures were available based on our criteria (described below and main article), our sample size was restricted to 250 peptide-HLA pairs. For testing consistency and stability, the number of peptides was mainly determined by computational cost and time to generate explanation. For consistency, we restricted to 200 peptides for 9 alleles each. This meant generating npeptides*nalleles*npredictors*nXAIs [200*9*2*2] explanations. Each explanation requires 25,000 model evaluations and therefore to test consistency we needed [200*9*6*2*25000] model evaluations. Increasing number of peptides would increase computational costs and

	therefore we limited to 200 peptides per allele. Similarly, for testing stability, we restricted to sampling 100 peptides from each cluster of peptides because it involves npeptides*nclusters*nXAIs*nevaluations [100*7*2*25000] model evaluations. Additionally, we had 6 cluster pairs. For each cluster pair, we calculated 3*100*100 euclidean distances for LIME and SHAP each. Increasing the number of peptides would have made the computation intractable.
Data exclusions	While the HLA alleles can bind to peptides ranging from length 8-15 amino acids, the most commonly observed length of peptides ligand is 9 amino acid. Hence we restrict our analysis to peptides of length 9. However, our code is designed to handle peptides of any length. Additionally, to ensure fairness in benchmarking MHC class I predictors, the MHC-Bench dataset excludes peptide-HLA pairs that were present in the training datasets of the respective predictors. Correspondingly, we only included PDB structures of bound peptide-HLA to peptides of length 9. In case of multiple PDB structures for a pair of peptide and HLA, PDB structures with finer resolution were selected. This is because the free energy calculations are affected by the resolution of the structures.
Replication	We provide the code used in this study in a GitHub repository which can be used to replicate the results. The settings used and input data is also provided for exact replication of result in the repository.
Randomization	For testing consistency, 200 peptides were selected for each of the 9 HLA alleles considered randomly using Python's Random package. For testing Stability, binding peptides were clustered using GibbsCluster and 100 peptides were selected from each clusters randomly using Python's Random
Blinding	Blinding is not applicable as this study does not contain any data collection or work with clinical data.

Reporting for specific materials, systems and methods

We require information from authors about some types of materials, experimental systems and methods used in many studies. Here, indicate whether each material, system or method listed is relevant to your study. If you are not sure if a list item applies to your research, read the appropriate section before selecting a response.

Materials & experimental systems

n/a	Involved in the study
☒	☐ Antibodies
☒	☐ Eukaryotic cell lines
☒	☐ Palaeontology and archaeology
☒	☐ Animals and other organisms
☒	☐ Clinical data
☒	☐ Dual use research of concern

Methods

n/a	Involved in the study
☒	☐ ChIP-seq
☒	☐ Flow cytometry
☒	☐ MRI-based neuroimaging
